# HLAtools, Searching Shared HLA Amino Acid Residue Prevalence, and the Global Frequency Browsers: New Computational Resources for Working With HLA Data and Visualizing Global Patterns of HLA Variation

**DOI:** 10.1111/iji.70013

**Published:** 2025-09-14

**Authors:** Livia Tran, Ryan Nickens, Vinh Luu, Effie W. Petersdorf, Steven J. Mack

**Affiliations:** ^1^ University of California San Francisco, Oakland California USA; ^2^ Lafayette College Easton Pennsylvania USA; ^3^ University of California Berkeley California USA; ^4^ Translational Science and Therapeutics Division, Fred Hutchinson Cancer Center Seattle Washington USA

**Keywords:** frequency distribution, global frequency browser, heatmap, HLA, HLAtools, informatics, SSHAARP

## Abstract

The highly polymorphic HLA genes inform susceptibility and resistance to infectious and autoimmune diseases and cancers and are key for successful solid‐organ and stem‐cell transplantation therapies. Over 41,000 HLA alleles are known and are unevenly distributed across the human population. Here, we describe HLAtools, Searching Shared HLA Amino‐Acid Residue Prevalence (SSHAARP) and the Global Frequency Browser (GFB), new informatic tools developed to facilitate working with HLA data and visualizing the global distribution of HLA variants in human populations. HLAtools is an R package that consumes static resources for HLA alleles and sequences and makes these data locally computable alongside data‐query, data‐customization and data‐analysis functions. The package further includes new reference datasets that dissect and catalogue HLA regions and HLA gene structures and provide insight into the organization of HLA pseudogenes and gene fragments. SSHAARP is an R package that describes the frequency distributions of individual HLA haplotypes, alleles and amino‐acid motifs as global heatmaps. Allele frequency maps for more than 800 HLA alleles can be browsed using the GFB web and mobile applications. HLAtools and SSHAARP are available from the Comprehensive R Archive Network, and the GFB apps are available on GitHub.

**Abbreviations**
AFND: Allele Frequency Net Database
ANHIG: Anthony Nolan HLA Informatics Group
CRAN: Comprehensive R Archive Network
GFB: Global Frequency Browser
GL: Genotype List
GMT: Generic Mapping Tools
IMGT: ImMunoGeneTics
IPD: ImmunoPolymorphism Database
KIR: Killer‐cell Immunoglobulin‐like Receptor
PALM: Population Allele Locating Mapmaker
pould: Phased Or Unphased Linkage Disequilibrium
SSHAARP: Searching Shared HLA Amino‐Acid Residue Prevalence

## 1 Introduction

HLA genes encode key elements of the adaptive immune system that enables the discrimination of self‐ and non‐self‐peptides through interactions with T cells and NK cells, respectively (Zinkernagel and Doherty 1974). Class I HLA proteins are expressed on all nucleated cells and present endogenous peptides to CD8+ T cells (Rosenstein et al. 1989); specific class I HLA alleles serve as ligands for the killer‐cell immunoglobulin‐like receptors (KIR) (Shin et al. 1999). Class II HLA proteins are expressed on immune monitoring cells and present exogenous peptides to CD4+ T cells (Doyle and Strominger 1987). Given these roles, HLA genes are highly relevant for understanding and intervening in infectious (Hill et al. 1991; Kaslow et al. 1990) and autoimmune diseases (W. Bodmer 1981, Gough and Simmonds 2007) and cancers (Wank and Thomssen 1991, Smith et al. 1989). Accurate identification of an individual's HLA genotype is key for successful stem‐cell and solid‐organ transplantation therapy (Park et al. 1988, Aversa et al. 1998, Martins et al. 2007). The notably uneven distribution of HLA variants across the human population makes these genes central for studies of population genetics and evolutionary biology (W. F. Bodmer and Thompson 1977, Solberg et al. 2008).

The HLA region (6p21.3) is the most polymorphic in the human genome (Robinson et al. 2000). As of July 2025, 42,193 unique nucleotide sequence variants have been identified for 45 HLA genes (D. J. Barker et al. 2023). Nomenclature and sequence information for HLA genes and their allelic variants is managed by the World Health Organization's Nomenclature Committee for Factors of the HLA System, with major nomenclature modifications effected in 2003 and 2010 (WHO Nomenclature Committee 1968).

HLA allele‐name and allele‐sequence data are housed in the ImmunoPolymorphism Database‐ImMunoGeneTics/HLA (IPD‐IMGT/HLA) Database (www.ebi.ac.uk/ipd/imgt/hla/), which has been updated quarterly since 1998 (Robinson et al. 2024). Protein, coding nucleotide and genomic alignments for HLA genes (as well as the *HFE*, *MICA*, *MICB*, *TAP1* and *TAP2* genes) in the last 62 quarterly IPD‐IMGT/HLA updates are maintained and made available by the Anthony Nolan HLA Informatics Group (ANHIG) on the ANHIG/IMGTHLA GitHub repository (github.com/ANHIG/IMGTHLA), along with documentation of HLA allele names going back to January of 2000 (Marsh et al. 2005). While these sequence and historical nomenclature resources are central for immunogenetics research and therapeutic applications, they are largely made available as static‐text files intended for human consumption, and as such preclude individualized queries to support broad applications across diverse biomedical models. An unmet need is a standardized, computable resource that can be consumed in an automated fashion, allowing these resources to be investigated via complex queries, and dissected and reformatted for user‐defined applications.

For example, allele sequence data in the ANHIG/IMGTHLA GitHub repository are available in multiple formats—MSF (Genetics Computer Group 1994), FASTA (Fuchs et al. 1990), PIR (W. C. Barker et al. 1999), xml (Bray et al. 1998), an EMBL‐ENA‐based flat file (Baker et al. 2000), and a text format that we refer to here as the ‘IMGT’ format (Robinson et al. 2024) (e.g., raw.githubusercontent.com/ANHIG/IMGTHLA/refs/heads/Latest/alignments/C_nuc.txt. Of these, only the IMGT format explicitly defines the start, end and gene‐feature boundary positions relative to a reference allele in a multiple sequence alignment format, making these alignments the most informative. While the IMGT format maximizes informative sequence content, it presents sequence data in blocks of 50–100 positions. In order to effectively apply these data, they must be converted to a table format, in which each allele sequence is on a single row, and all the variants for a given position are in a single column. Although the BIGDAWG R package (Pappas et al. 2016) performs this conversion for IMGT‐format protein alignments, it does not support nucleotide, codon or genomic alignments, nor the conversion of protein alignments of other IPD‐IMGT/HLA Database genes. A general‐purpose system designed to consume IPD‐IMGT/HLA Database resources and convert them into computable data objects is lacking.

The frequencies of HLA alleles and haplotypes continue to be an area of intense biomedical interest both from the perspective of mechanistic studies that unravel the functional implications of HLA diversity, as well as clinical applications in infectious diseases, autoimmunity and transplantation. While the IPD‐IMGT/HLA Database is a key resource for HLA nomenclature and allele sequence data, it provides little information on the frequencies of HLA alleles and haplotypes in populations. In 2008, Solberg et al. (2008) described a meta‐analysis dissecting natural selection and heterogeneity in the class I and class II HLA alleles in a global sample of 66,800 individuals in 497 populations, based on the global allele‐frequency distributions of over 800 two‐field *HLA‐A*, *HLA‐B*, *HLA*‐*C*, *HLA‐DRB1*, *HLA‐DQA1*, *HLA‐DQB1*, *HLA‐DPA1* and *HLA‐DPB1* alleles. Solberg et al. published a frequency heatmap describing the global allele frequency distribution of the DRB1*15:01 allele in 225 non‐migrant populations and made frequency heatmaps for 804 other alleles available alongside the allele frequency data at www.pypop.org/popdata. While these maps provide insight into the global distribution (or lack thereof) of high‐frequency HLA alleles, Solberg et al. provided only a 20‐line shell script to enable replication or generation of new maps.

The Allele Frequencies Net Database (AFND; www.allelefrequencies.net) is a larger resource that houses over 156,000 HLA allele and haplotype frequencies for 1324 population studies, along with frequency data for *KIR*, MHC class I chain‐related (*MIC*) genes and cytokine polymorphisms (Gonzalez‐Galarza et al. 2024). The AFND enables structured queries of classical and non‐classical HLA allele and haplotype frequencies, as well as the generation of maps identifying the frequency of a given allele in each population. While AFND frequency data are easily viewed, it is difficult to gain direct access to these data for third‐party applications.

Finally, we note that the focus of Solberg et al., AFND, and the immunogenetics community in general, has been on the frequency distributions of alleles and their haplotypes. However, it has been well documented that specific amino acid variants and variant motifs, often shared across individual HLA alleles, can have clinical (Fang et al. 2021) and evolutionary (Single et al. 2024) significance. The global frequency distributions of these motifs can potentially be more revelatory than the frequencies of the individual alleles that encode them, and a system to visualize them in an automated manner could be impactful.

To address these unmet needs, we describe three new software applications for working with HLA data and visualizing the global frequency distributions of HLA alleles, haplotypes and amino‐acid motifs—the HLAtools and Searching Shared HLA Amino‐Acid Resident Prevalence (SSHAARP) R packages, and the Global Frequency Browser (GFB) applications. HLAtools (v1.6.3) and SSHAARP (v2.0.8) are R packages that interact directly with IPD‐IMGT/HLA Database resources and are available from the Comprehensive R Archive Network (CRAN; cran.r‐project.org). Web and mobile‐device versions of the GFB applications (versions 1.0.4 and 1.2.3, respectively) are available as GitHub Pages.

## 2 Methods

### 2.1 Development of HLAtools

Version 1.6.3 of the HLAtools R package was developed in the R statistical computing environment (version 4.4.2, www.r‐project.org) (R Core Development Team 2013), using version 2025.05.0+496 of the RStudio Desktop integrated development environment (www.rstudio.com) (RStudio Team 2020), and is released under a GPL (≥3) license. The current version of HLAtools is available on CRAN at cran.r‐project.org/package=HLAtools and can be installed in the R environment using the command *install.packages(‘HLAtools’)*. Developmental versions of HLAtools are available at github.com/sjmack/HLAtools. HLAtools’ datasets and functions are documented in the package, which includes a vignette detailing them, along with examples of their use. The HLAtools vignette is included here as Supporting Information 1. An overview of the package, describing key datasets and functions, with examples of their applications, can be found at github.com/sjmack/HLAtools/.

### 2.2 HLAtools Datasets

HLAtools makes computable versions of text‐based online resources available alongside a suite of search, query and data‐analysis functions, enabling these data to be queried, dissected and transformed for user‐defined purposes. The package includes nine datasets. Four of these (*GLstring.ex*, *GLSC.ex*, *UNIFORMAT.example* and *sHLAdata*) are derived from existing resources with minimal modifications.

The *GLstring.ex* dataset was derived from the *hla.hap.demo* dataset in the Phased or Unphased Linkage Disequilibrium (pould) R package (version 1.0.1; CRAN.R‐project.org/package=pould) (Osoegawa et al. 2019).

The GL String Codes (Mack, Sauter, et al. 2023) in *GLSC.ex* were built from the GL Strings (Mack, Schefzyk, et al. 2023) in *GLstring.ex* and assigned under IPD‐IMGT/HLA Database version 3.01.0.

The *UNIFORMAT.example* dataset was derived from the ‘Example of a file for 3 loci HLA data’ document at hla‐net.eu/wp/wp‐content/uploads/example‐three‐loci.unif_.txt (Nunes 2007).

The *sHLAdata* (‘synthetic HLA data’) dataset was originally developed as part of the 13th International HLA Workshop (Mack et al. 2007) for demonstrating PyPop (Lancaster et al. 2024) (pypop.org) functions and is derived from raw.githubusercontent.com/alexlancaster/pypop/refs/heads/main/tests/data/USAFEL-UchiTelle.pop, with the first two lines and the ‘dra’ columns of the original dataset excluded. These synthetic HLA data were translated from IPD‐IMGT/HLA Database release version 2.08.0 to version 3.56.0 via HLAtools’ GIANT() function, and do not represent data for real individuals.

**FIGURE 1:**
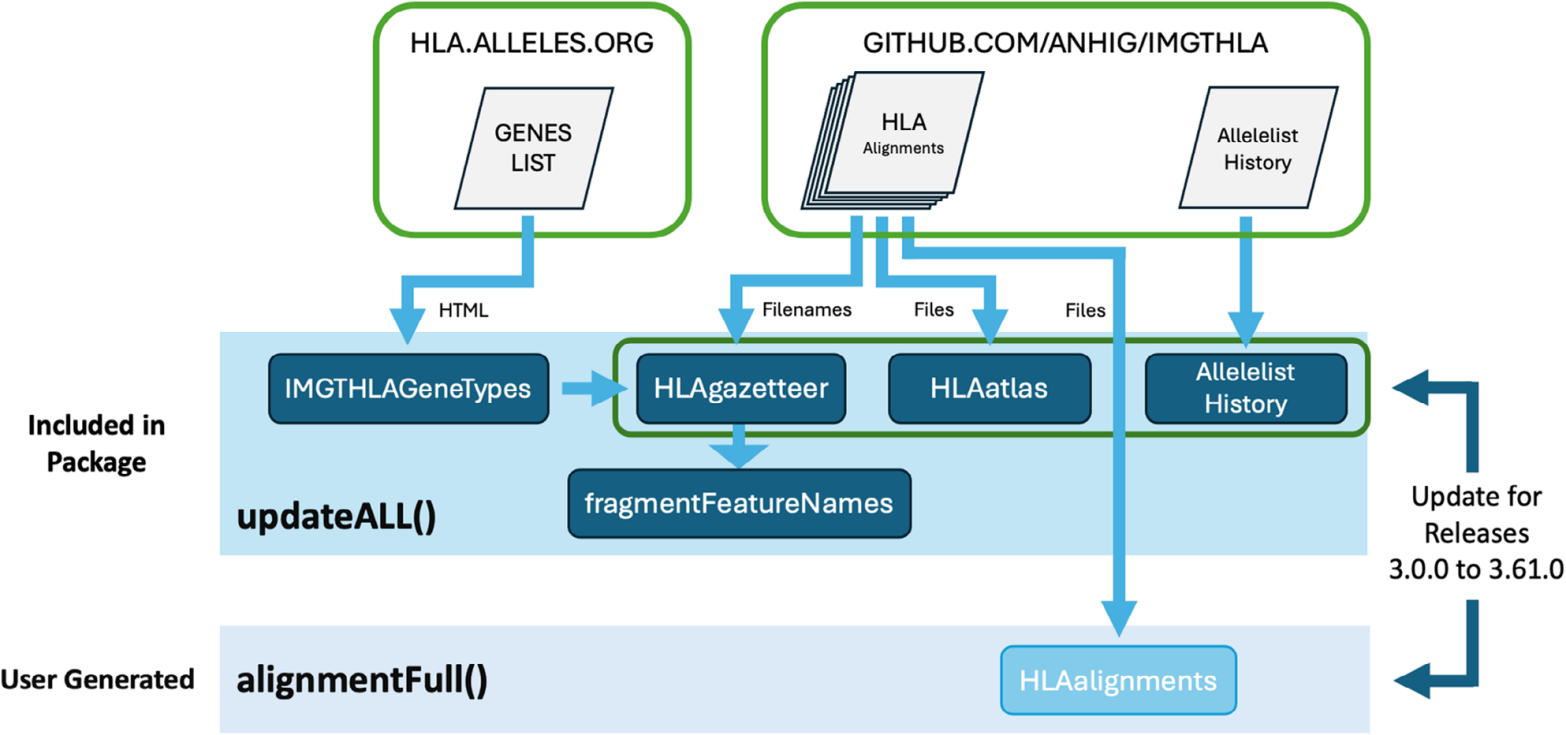
Cartoon workflow depicting the construction of package datasets. Each of the six constructed datasets are built by an individual HLAtools function. As illustrated, these functions depend on data resources available on either the hla.alleles.org website or the ANHIG/IMGTHLA GitHub repository.

Five packaged datasets (*alleleListHistory*, *IMGTHLAGeneTypes*, *fragmentFeatureNames*, *HLAatlas* and *HLAgazetteer*), as well as the *HLAalignments* dataset, are built de novo by package functions and can be updated as necessary with successive IPD‐IMGT/HLA Database releases. Each dataset includes a *version* element that identifies the version of the source data use to generate it. Figure 1 illustrates the data resources and workflows used to construct these datasets.

As illustrated in Figure 1, the *alleleListHistory* dataset is built using the buildAlleleListHistory() function, which processes the allelellist_history.txt file on the ANHIG/IMGTHLA GitHub repository. The *fragmentFeatureNames* dataset is built using the ffN() function, which extracts the names of gene fragments and pseudogenes from the *HLAgazetteer* dataset. The *HLAatlas* dataset is built using the atlasMaker() function, which processes sequence alignment files. The *HLAgazetteer* dataset is built using the buildGazetteer() function, which processes the names of sequence alignment files. The *IMGTHLAGeneTypes* datset is built using the buildIMGTHLAGeneTypes() function, which processes HTML data on the hla.alleles.org webpage. The *HLAalignments* dataset is built using the alignmentFull() function, which processes the same alignment files as atlasMaker().

While each function can be called independetly, the five functions above are called by the updateAll() function, allowing all five package datasets to be rebuilt with each IPD‐IMGT/HLA Database release via one command. Using updateAll(), the *alleleListHistory*, *HLAatlas* and *HLAgazetteer* datasets can be built from the source files on any ANHIG/IMGTHLA GitHub repository branch (each branch corresponds to an IPD‐IMGT/HLA Database release). However, as there is only one publically available version of the hla.alleles.org website, the *IMGTHLAGeneTypes* and *fragmentFeatureNames* datasets cannot be reverted to earlier versions.

The alignmentFull() function will build all available protein, coding nucleotide, codon and genomic alignments in a specified ANHIG/IMGTHLA GitHub repository branch, defaulting to the current branch. With a compressed size of 24.2 MB, the *HLAalignments* dataset is too large to be included with the HLAtools package, and must be built by the user, by executing *HLAalignments <‐ alignmentFull()*, after the package has been installed.

Individual alignments for specific genes and alignment types can be built using the buildAlignments() function. In this case, the returned dataset must be named ‘HLAalignments’, and must follow the *HLAalignments* dataset's internal structure, in order to be available to package functions.

As with *HLAalignments*, the *alleleListHistory* datasets built for IPD‐IMGT/HLA Database release versions 3.60.0 and later are too large to be included with the HLAtools package. Versions of these datasets for later releases must be built after the package has been installed. The *alleleListHistory* dataset included with the HLAtools pacakge was built for IPD‐IMGT/HLA Database release 3.59.0. The remaining constructed datasets were built under IPD‐IMGT/HLA Release 3.61.0.

Three of these datasets are completely novel and were created to provide insight into the structure and organization of IPD‐IMGT/HLA Database genes.

The *fragmentFeatureNames* dataset identifies the standard and non‐standard gene features (exons, introns and untranslated regions [UTRs]) of the *HLA‐DPA2*, *HLA‐DPB2*, *HLA‐H*, *HLA‐J*, *HLA‐K*, *HLA‐L*, *HLA‐N*, *HLA‐P*, *HLA‐R*, *HLA‐S*, *HLA‐T*, *HLA‐U*, *HLA‐V*, *HLA‐W* and *HLA‐Y* gene fragments and pseudogenes. For gene feature comparisons, the *HLA‐DPA2* and *HLA‐DPB2* pseudogenes were aligned relative to the *HLA‐DPA1* and *HLA‐DPB1* genomic alignments, respectively. The class I pseudogenes and gene fragments were aligned relative to the *HLA‐C* genomic alignment, as the gene body, the contiguous sequence of all exons and introns, for *HLA‐C* (26,295 nucleotides) is longer than the gene bodies of *HLA‐A* (26,150) or *HLA‐B* (20,189). For each gene, a features element, identifying standard and non‐standard gene features, and an annotation element, providing detail and context for specific features, are included. An added version element identifies the pertinent IPD‐IMGT/HLA release version.

For each available type of alignment for each gene, *HLAatlas* identifies the position of each gene‐feature boundary in that alignment.

*HLAgazetteer* organizes the named genes of the HLA region into 19 structural and functional categories.

### 2.3 Development of SSHAARP

The SSHAARP package generates global frequency heatmaps for HLA alleles, user‐defined HLA haplotypes and user‐defined HLA amino‐acid motifs.

Version 2.0.8 of the SSHAARP package was developed in the R statistical computing environment (version 4.4.1), using version 2024.09.0+375 of the RStudio Desktop integrated development environment (www.rstudio.com), and is released under a GPL (≥ 3) license. SHAARP applies Generic Mapping Tools (Wessel et al. 2019) (GMT) version 6.5.0 (www.generic‐mapping‐tools.org), the gmt (Magnusson 2022) R package (CRAN.R‐project.org/package=gmt) version 2.0.3 and Ghostscript (ghostscript.com/releases/gsdnld.html) version 10.04.0 to generate heatmaps. GMT and Ghostscript must be installed in the local operating system before SSHAARP can be applied to generate maps. The current version of SSHAARP is available on CRAN at CRAN.R‐project.org/package=SSHAARP and can be installed in the R environment using the command *install.packages(‘SSHAARP’)*. Developmental versions of SSHAARP are available at github.com/liviatran/SSHAARP_package. SSHAARP's datasets and its primary functions are documented in the package, which includes a vignette detailing them, along with examples of their use. The SSHARRP vignette is included here as Supporting Information 2.

### 2.4 SSHAARP Datasets

SSHAARP includes two datasets. The *solberg_dataset* dataset comprises frequency, latitude and longitude data for 840 two‐field *HLA‐A* (165 alleles), *HLA‐B* (295 alleles), *HLA‐C* (77 alleles), *HLA‐DRB1* (189 alleles), *HLA‐DQA1* (8 alleles), *HLA‐DQB1* (30 alleles), *HLA‐DPA1* (9 alleles) and *HLA‐DPB1* (67 alleles) locus alleles in a global sample of 66,830 individuals in 497 populations (pypop.org/popdata/2008/data.html) (Solberg et al. 2008). The names of two alleles in *solberg_dataset* have changed since they were initially named. C*03:12 was changed to C*03:307 in 2015, and DQB1*02:03:01 was changed to DQB1*02:180 in 2020. These allele names have been updated in *solberg_dataset*. The names of the A*26:11, B*13:07, B*51:27, B*51:44 and DPB1*61:01 alleles, which had previously been presented sans expression variants in *solberg_dataset*, were extended to A*26:11N, B*13:07N, B*51:27N, B*51:44N and DPB1*61:01N. In addition, the C*05:01/C*05:09 ambiguity in the Lebanese_2006 population was changed to C*05:01 in *solberg_dataset*.

The *mock_haplotype_dataset* dataset is a synthetic dataset provided for demonstrating the generation of haplotype maps. It includes *HLA‐A∼HLA‐B∼HLA‐C∼HLA‐DRB1* haplotypes for 47,214 subjects in 21 synthetic populations. This dataset follows the haplotype format used by AFND, which houses frequency data for the *HLA*, *KIR*, *MIC* and cytokine genes (Santos et al. 2016).

SSHAARP's PALM() (‘Population Allele Locating Mapmaker’) function applies HLAtools’ buildAlignments() function to build the protein alignments that are used to generate amino‐acid motif, allele and haplotype frequency heatmaps. These alignments are stored in the tempdir() folder for a given R session, on a per locus basis. Given this, the first time a map for a specific locus is generated in an R session, the generation of that map will take longer than subsequent maps.

### 2.5 Development of the Web and Mobile GFB Applications

Version 1.0.4 of the web GFB application and version 1.2.3 of the mobile GFB application were developed using CSS, HTML and JavaScript in Visual Studio Code version 1.9.10 (github.com/microsoft/vscode). The individual allele frequency maps presented in the GFB applications were generated by applying SSHAARP's PALM() function to the alleles in *solberg_dataset*, with the *filterMigrant* parameter set to TRUE to prevent the generation of maps for migrant or admixed populations, and the low frequency parameter (*generateLowFreq*) set to TRUE to enable the generation of maps for alleles with low frequencies (<0.0001). Of the 840 HLA alleles in *solberg_dataset*, 35 were observed solely in migrant or admixed populations and were excluded. The changes and extensions to allele names described in Section 2.4 are reflected in the GFBs. The resulting 805 maps are available at github.com/liviatran/pypopMaps. The web and mobile GFBs are available at globalfrequencybrowser.github.io/GFB/webapp/ and globalfrequencybrowser.github.io/GFB/mobileapp/, respectively.

## 3 Results

HLAtools, SSHAARP and the GFBs are new applications developed for HLA informatics and research. HLAtools functions in R version 3.6.0 and higher and includes 72 functions that depend on the DescTools (Signorell 2025), dplyr (Wickham et al. 2023), fmsb (Nakazawa 2024), rvest (Wickham 2024), stats (R Core Development Team 2013), stringr (Wickham 2023), tibble (Müller 2023), utils (R Core Team 2024) and xfun (Xie 2024) R packages (which have their own dependencies), and nine datasets, five of which can be updated with each IPD‐IMGT/HLA Database release. A tenth dataset can be built after the package is installed. HLAtools’ functions and datasets are documented in the package, and detailed in Supporting Information 1.

SSHAARP functions in R version 3.6.0 and higher and includes 17 functions that depend on the data.table, DescTools (Signorell 2025), dplyr (Wickham et al. 2023), utils (R Core Team 2024), filesstrings (Nolan and Padilla‐Parra 2017), gmt (Magnusson 2022), gtools (Warnes et al. 2023), HLAtools, purrr (Wickham 2023), stringi (Gagolewski 2022) and stringr (Wickham et al. 2023) R packages (which have their own dependencies), as well as two datasets. As with HLAtools, SSHAARP's functions and datasets are documented in the package. SSHAARP's vignette detailing the use of the package's primary PALM() function is provided as Supporting Information 2.

The GFB apps are client‐side web applications that enable the browsing and side‐by‐side comparison of colour frequency heatmaps, for 805 *HLA‐A*, *HLA‐C*, *HLA‐B*, *HLA‐DRB1*, *HLA‐DQA1*, *HLA‐DQB1*, *HLA‐DPA1*, and *HLA‐DPB1* alleles, which were generated by SSHARP. Overviews of HLAtools, SSHAARP and the GFBs, along with key datasets and functions, are provided below.

### 3.1 New Reference Datasets in the HLAtools R Package

Many of HLAtools’ functions interact with reference datasets or require reference datasets to operate. The *HLAgazetteer*, *fragmentFeatureNames* and *HLAatlas* datasets are new reference datasets that summarize, catalogue and define extant IPD‐IMGT/HLA resources.

*HLAgazetteer* organizes the genes curated by the IPD‐IMGT/HLA Database into 19 informative categories (elements). For example, *HLAgazetteer*’s *align* element identifies all of the genes for which any alignments exist, and the *prot* element identifies all of the genes with protein alignments. The *map* element identifies all of the named genes of the HLA region curated by the IPD‐IMGT/HLA Database in their centromeric to telomeric chromosomal map order. This element includes the recently named *HLA‐R* gene (Alexandrov et al. 2023), which occurs with *HLA‐Y* on a 60 kb chromosomal insertion variant (Figure S1). The map order of the *HLA‐DRB3*, *DRB4* and *DRB5* genes, and *DRB6* and *DRB7* genes is ambiguous, due to structural (Andersson 1998) and length variation in the DRB region (Figure S2). The *DRB3*, *DRB4* and *DRB5* genes are identified as ‘DRB3/4/5’ and the *DRB6* and *DRB7* genes as ‘DRB6/7’ in the *map* element.

*HLAgazetteer* is used to build *fragmentFeatureNames*, which identifies and annotates the standard and novel gene features of pseudogenes and gene‐fragments. While expressed genes comprise exons, introns and untranslated regions, the structures of pseudogenes and gene‐fragments are less constrained, and do not always correspond to those of expressed genes. Based on the gene‐feature boundaries in the genomic alignments of pseudogenes and gene‐fragments, *fragmentFeatureNames* identifies non‐standard features described as *hybrid* (H), *join* (J), *novel* (N) or *segment* (S) features. A *hybrid* feature includes nucleotide sequence that aligns to a feature in the genomic reference, as well as sequence that does not align to the reference. A *join* feature incudes contiguous sequence from two or more features that are separated by a gene‐feature boundary in the genomic reference. A *novel* feature does not correspond to a known genomic reference feature. A *segment* feature is a subset of a longer feature sequence in the genomic reference. Each annotation of a pseudogene or gene fragment in *fragmentFeatureNames* provides a description of these novel features.

Each feature is numerically suffixed to identify its order of appearance in the 5’ to 3’ direction. For example, features for the *HLA‐R* gene fragment are H.1, E.3, I.3, E.4, I.4, E.5, I.5, E.6, I.6, J.1 and J.2, indicating that sequence features Exon 3 through Intron 6 are present in *HLA‐R*, flanked by non‐standard features that are not present in the *HLA‐C* genomic reference. The annotation for non‐standard *HLA‐R* features is, ‘*H.1 is 287 nucleotides of novel sequence followed by 156 nucleotides of the 5'end of Intron 2, which includes a 5‐nucleotide section of novel sequence. J.1 is all of Exon 7 and the first 12 nucleotides of Intron 7. J.2 is the remainder of Intron 7, and the 3' UTR. Exon 8 is absent*’.

*fragmentFeatureNames* is used to build *HLAatlas*, which identifies the location of gene‐feature boundaries in each type of alignment for each aligned gene in a prot, nuc, or gen element relative to a specific nucleotide, codon or peptide position. For example, the *HLAatlas$nuc$G* element indicates that the exon 3–exon 4 boundary in the *HLA‐G* nucleotide alignment is between nucleotides 619 and 620, and in codon 183. *HLAatlas* applies the non‐standard gene feature nomenclature for pseudogene and gene fragment atlases where necessary.

### 3.2 Computable Versions of Existing Reference Datasets

A key goal in developing *HLAtools* was to make static, text‐based data resources computable, allowing them to be searched, queried and applied for new purposes.

*IMGTHLAGeneTypes* summarizes molecular characteristics of HLA region genes and informs the construction of *HLAgazetteer*. *AlleleListHistory* informs HLAtools’ translateGLstring(), updateGL() and GIANT() functions, enabling translation of individual HLA alleles, HLA GL Strings, HLA GL String Codes and entire HLA datasets across IPD‐IMGT/HLA release versions.

The verifyAllele() and queryRelease() functions apply *alleleListHistory* to confirm HLA allele names, identify which releases specific allele names occurred in and identify allele names that share components in a specific release.

*HLAalignments* makes all of the individual protein, nucleotide and genomic alignments in a given IPD‐IMGT/HLA Database release version available for structured searches and queries of allele names, user‐defined sequence motifs and variant positions via the validateAllele(), alignmentSearch() and queryPositions() functions, and enables the construction of customized, multi‐locus alignments via the customAlign() function. Details and examples of these data objects and functions are provided in the HLAtools vignette (Supporting Information 1).

### 3.3 Generating HLA Frequency Heatmaps With the SSHAARP R Package

**FIGURE 2:**
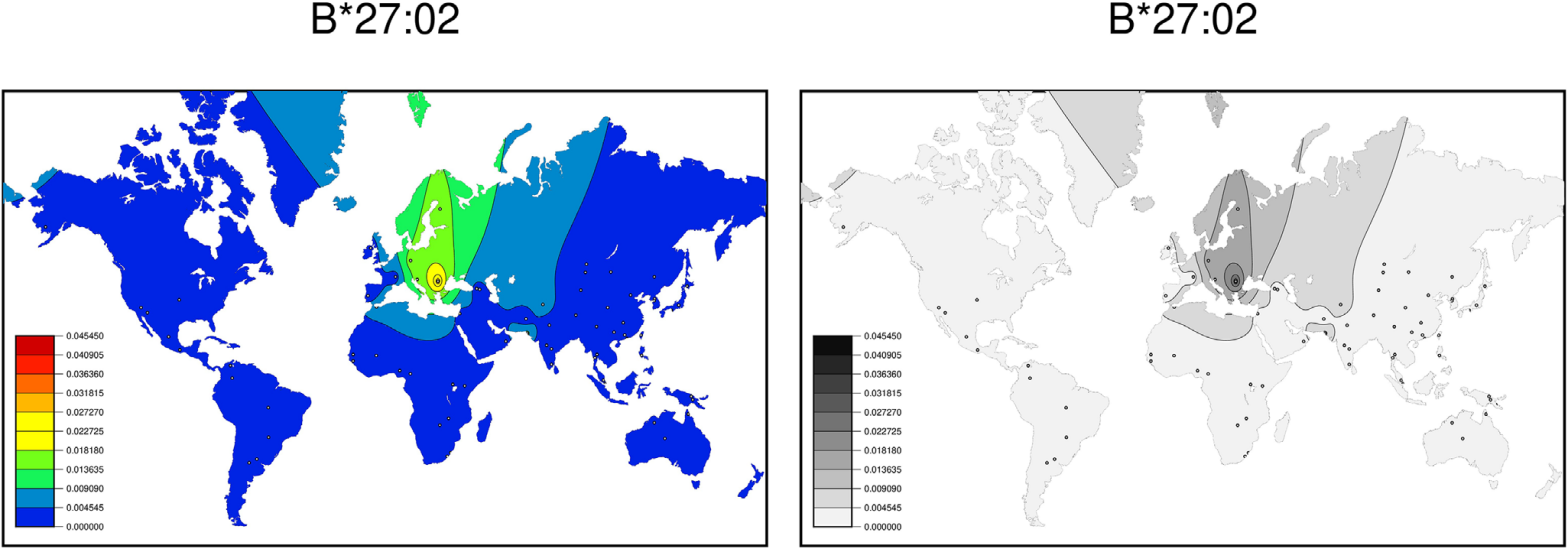
Colour and greyscale global heatmaps for the HLA‐B*27:02 allele. Each white dot indicates an indigenous population for which HLA‐B allele frequency data were available in SSHAARP's *solberg_dataset*. The frequency of this allele is indicated by the colour range from blue (lowest) to red (highest) in the left (colour) pane, and from white (lowest) to black (highest) in the right (greyscale) pane. The highest observed frequency for the HLA‐B*27:02 allele is 0.045 in eastern Europe.

SSHAARP is more narrowly focused than HLAtools; its main purpose is to generate frequency heatmaps for HLA alleles (Figure 2), amino‐acid motifs and haplotypes. While the maps presented reflect the frequency distributions in *solberg_dataset* and *mock_haplotype_dataset*, third‐party frequency datasets that follow the *solberg_dataset* and *mock_haplotype_dataset* structures can be applied to generate maps for variants at any locus supported in the ANHIG/IMGTHLA GitHub repository, including non‐HLA loci. Details and examples of these data objects and SSHARRP's functions are provided in the SSHAARP vignette (Supporting Information 2).

**FIGURE 3:**
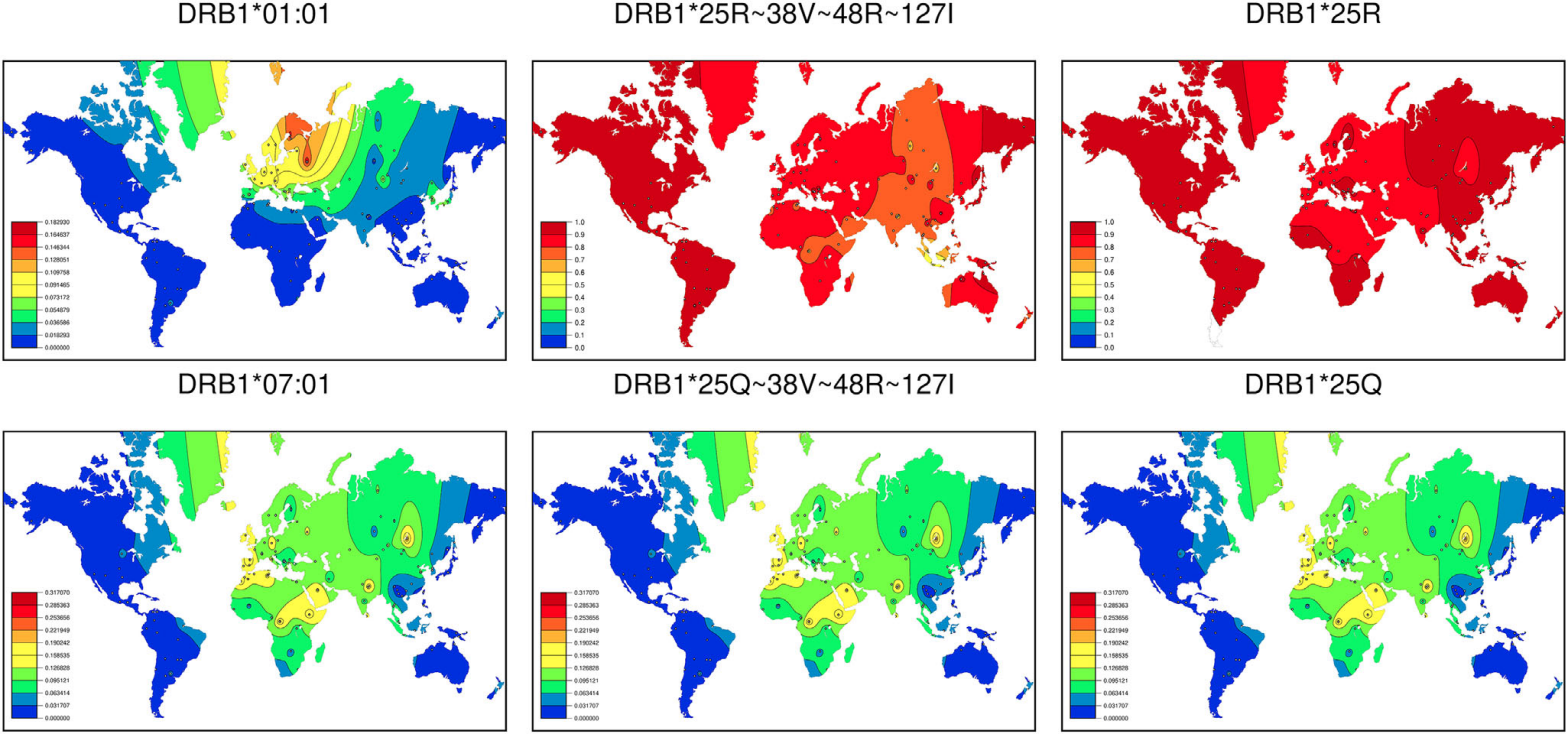
Investigating global allele and amino acid motif distributions. Colour frequency heatmaps for two *HLA‐DRB1* alleles (left), two four‐residue DRB1 amino‐acid motifs (centre) and two DRB1 amino acid residues (right) are shown. When there are no population data in regions near the borders of a heatmap, the ‘clines’ for a given variant may not extend into that area, resulting in a white region of landmass, as with the southern tip of South America in the map for the DRB1*25R motif.

Despite SSHAARP's narrow focus, HLAtools functions can be applied to expand its utility. Figure 3 illustrates the use of HLAtools’ alignmentSearch() function to compare the global frequency distributions of HLA alleles to the frequencies of their constituent amino‐acid motifs. In this figure, the heatmaps on the left, for DRB1*01:01 and DRB1*07:01, were generated by specifying each allele name in SSHAARP's PALM() function (e.g., PALM(“DRB1*01:01:01:01”, variantType = “allele”, filename = solberg_dataset)), while the amino acid motif heatmaps were generated using HLAtools' alignmentSearch() function to determine the amino‐acid variants at the same positions in each allele, without a priori knowledge of the sequence at those positions (e.g., PALM(variant = paste(“DRB1”, alignmentSearch(“prot”, “DRB1*01:01:01:01”, c(25, 38, 48, 127)), sep = “*”), “motif”, filename = solberg_dataset)). In this case, alignmentSearch() extracted the motif from the DRB1*01:01 allele, and PALM() identified all alleles at the *DRB1* locus that share that motif.

The resulting amino acid motif heatmaps describe the distribution of the combined frequencies of all alleles in *solberg_dataset* that share a specific motif. The heatmaps in the centre of Figure 3 indicate that the DRB1 amino acid residues at positions 25, 38, 48 and 127 in DRB1*01:01 and DRB1*07:01 differ only at amino acid position 25. HLAtools’ motifMatch() function reveals that the DRB1*25R∼38V∼48R∼127I motif is observed in 976 2‐field *DRB1* alleles in IPD‐IMGT/HLA Database release 3.59.0, and thereby in 65% of the *DRB1* alleles in *solberg_dataset*, so that the DRB1*25R∼38V∼48R∼127I motif is observed at frequencies greater than 0.7.

Conversely, motifMatch() reveals that the DRB1*25Q∼38V∼48R∼127I motif is observed in 86 two‐field *DRB1* alleles, only two of which (DRB1*01:13 and DRB1*13:92) are not DR7 alleles. Only four of these 86 alleles (DRB1*07:01, DRB1*07:03, DRB1*07:07 and DRB1*07:04) are present in *solberg_dataset*, and while some DRB1*07 alleles encode other amino acid 25 residues (e.g., DRB1*07:50 encodes 25R and DRB1*07:114 encodes 25L), those alleles are not included in *solberg_dataset*. This is also true for alleles in other families that do not encode amino acid 25R (e.g., DRB1*08:26 encodes 25L). Finally, the heatmaps on the right indicate that the DRB1*25R motif is present on other 25∼38∼48∼127 motifs, resulting in higher overall frequencies for DRB1*25R relative to the DRB1*25R∼38V∼48R∼127I motif, while 25Q is restricted to DR7 alleles.

**FIGURE 4:**
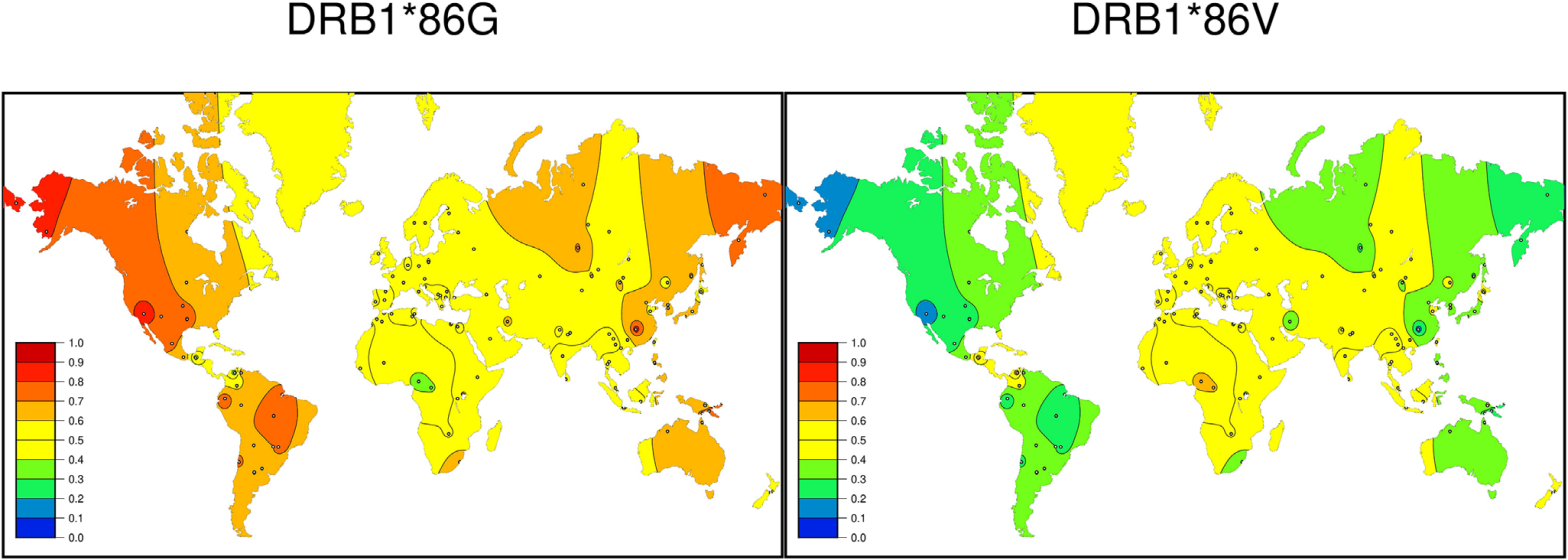
Global frequency heatmaps for DRβ position 86 residues. Colour heatmaps depicting the distributions of the DRβ‐86G and DRβ‐86V residues are shown. Both maps were generated by setting PALM()’s *mapScale* parameter to FALSE, so that the maximum frequency on both maps is 1.0, allowing direct comparison of the frequency distributions.

SSHAARP's motif heatmaps can have clinical applications. HLAtools’ queryPosition() function reveals that of the 3825 *DRB1* alleles in IPD‐IMGT/HLA Database release 3.60.0, 97% have a Gly or Val residue at DRβ position 86 (DRβ‐86). Variation at this DRβ position impacts post‐haploidentical related donor hematopoietic cell transplantation (HCT) disease relapse, with significantly lower risk of relapse among patients homozygous for DRβ‐86 Gly (GlyGly) whose mismatched related donors were heterozygous at DRβ‐86 (GlyVal) (Petersdorf et al. 2024). The global frequencies of the DRβ‐86 Gly and DRβ‐86 Val motifs are illustrated in Figure 4. The frequency scales of both maps range from 0% to 100%, allowing direct comparison of the frequencies of each variant. Based on each map, the frequency of DRβ‐86G or DRβ‐86 V homozygotes and DRβ‐86G/86 V heterozygotes, and the potential availability of a heterozygous donor for a homozygous patient, can be estimated for different world regions.

In indigenous North and South American populations, where the frequency of DRβ‐86G is no less than 0.4, and can be as high as 0.9, a large percentage of the population, between 65% and 80% of individuals in some areas, may be DRβ‐86G homozygotes; up to 48% may be DRβ‐86G/86V heterozygotes, and as few as 2%–4% may be DRβ‐86V homozygotes. Conversely in Central American, European and African populations, the frequencies of these variants are approximately 0.5, such that roughly 50% of individuals may be DRβ‐86G/86V heterozygotes, while individuals who are homozygous for either variant each represent only 25% of the population. For haploidentical related donor HCT therapy, it may be easier to find suitable DRβ‐86G/86V relatives for DRβ‐86 Gly HCT patients in African, Central American and European populations than in North and South American populations.

**FIGURE 5:**
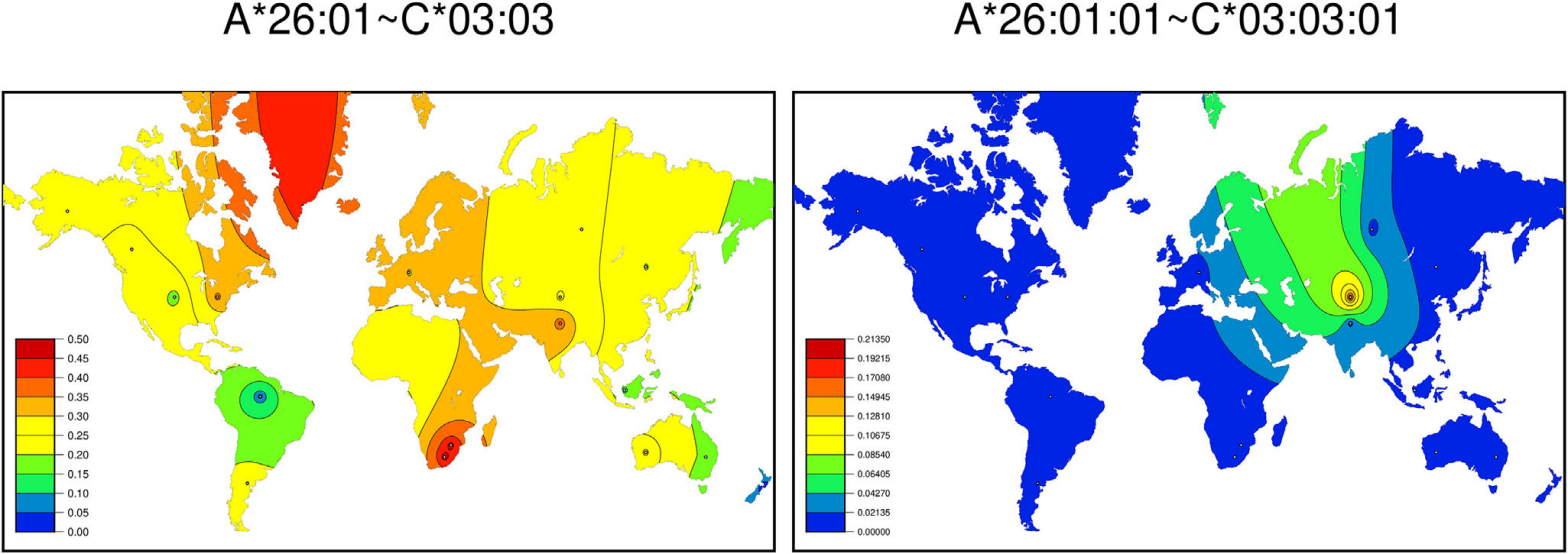
Generating haplotype maps at different allele name resolutions. Colour heatmaps depicting the frequency distributions of two‐field (left) and three‐field (right) versions of an *HLA‐A*∼*HLA‐C* haplotype in 16 populations are shown. These heatmaps were generated with synthetic data and do not represent the ‘real world’ distribution of this haplotype.

SSHAARP's *mock_haplotype_dataset* includes *A∼B∼C*, *A∼B∼DRB1* and *A∼B∼C∼DRB1* haplotypes for 21 synthetic populations. This dataset was created to represent haplotype data from multiple sources; haplotypes in 17 populations use two‐field allele names, and haplotypes in four populations use three‐field allele names. The individual genes specified can be extracted from longer, multi‐gene haplotypes, and truncated allele names can be extracted from longer allele names, to generate maps that maximize the utility of a dataset. This is illustrated in Figure 5, where the frequency range of the A*26:01∼C*03:03 haplotype is much larger and frequencies are generally much higher than for the A*26:01:01∼C*03:03:01 haplotype.

### 3.4 Browsing Global HLA Frequency Maps

HLAtools, SSHAARP and the GFBs constitute a pipeline that consumes static IPD‐IMGT/HLA Database and ANHIG/IMGTHLA GitHub repository resources, converts them into computable data objects, generates heatmaps describing the frequency distributions of HLA amino‐acid motifs, alleles and haplotypes, and makes heatmaps for common HLA alleles available for browsing. The web and mobile GFB applications are the endpoints of this pipeline.

**FIGURE 6:**
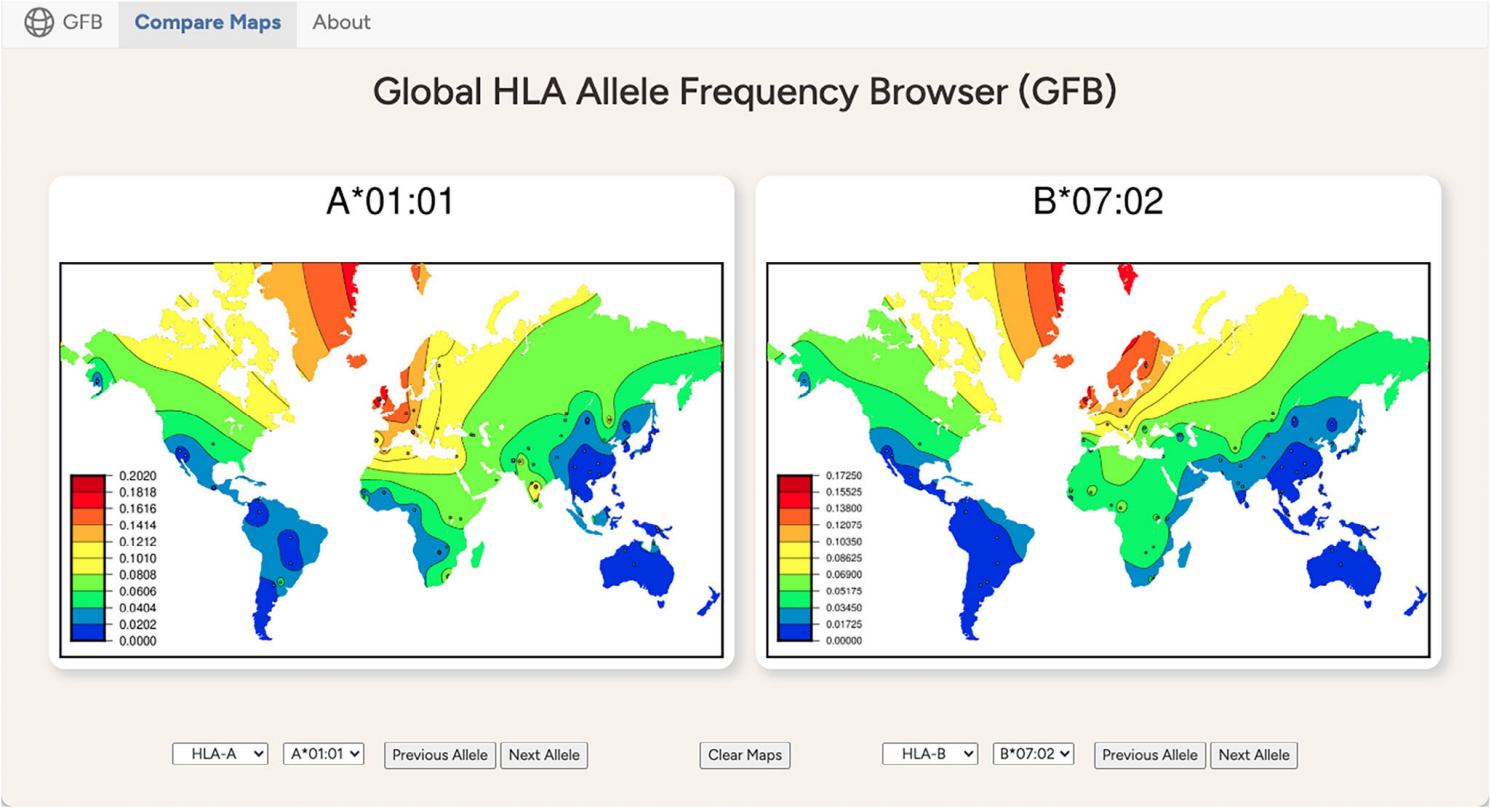
The Global Frequency Browser web application. The GFB web application enables side‐by‐side comparison of pairs of colour frequency heatmaps for 805 *HLA‐A*, *HLA‐C*, *HLA‐B*, *HLA‐DRB1*, *HLA‐DQA1*, *HLA‐DQB1*, *HLA‐DPA1* and *HLA‐DPB1* alleles. The browser consists of two tabs. The “Compare Maps” tab is the primary tab, containing the browser's controls—a set of locus and allele pulldowns and a pair of navigation buttons below each map pane. These pulldowns select the frequency map that will appear in that pane. The “Previous Allele” and “Next Allele” navigation buttons allow browsing through alleles in allele name‐order, and across HLA genes in map order, from class I to class II. The "Clear Maps" button will replace both maps with whitespace. A "Show Maps" button reveals the selected maps after maps have been cleared. Selecting a new allele will automatically display that allele. The "About" tab contains additional information about the GFB.

The GFB applications present colour frequency heatmaps generated using SSHAARP for the 805 *HLA* alleles observed in non‐migrant populations in *solberg_dataset*. Both applications require internet connectivity to access the frequency heatmaps. However, once a map has been viewed, it remains in the browser's cache and can be viewed offline. As illustrated in Figure 6, two maps from any of the *HLA‐A*, *HLA‐B*, *HLA‐C*, *HLA‐DRB1*, *HLA‐DQA1*, *HLA‐DQB1* and *HLA‐DPA1* loci can be viewed in the web GFB. A specific map can be selected by navigating locus and allele pulldowns, or maps at a given locus can be browsed sequentially by allele name.

**FIGURE 7:**
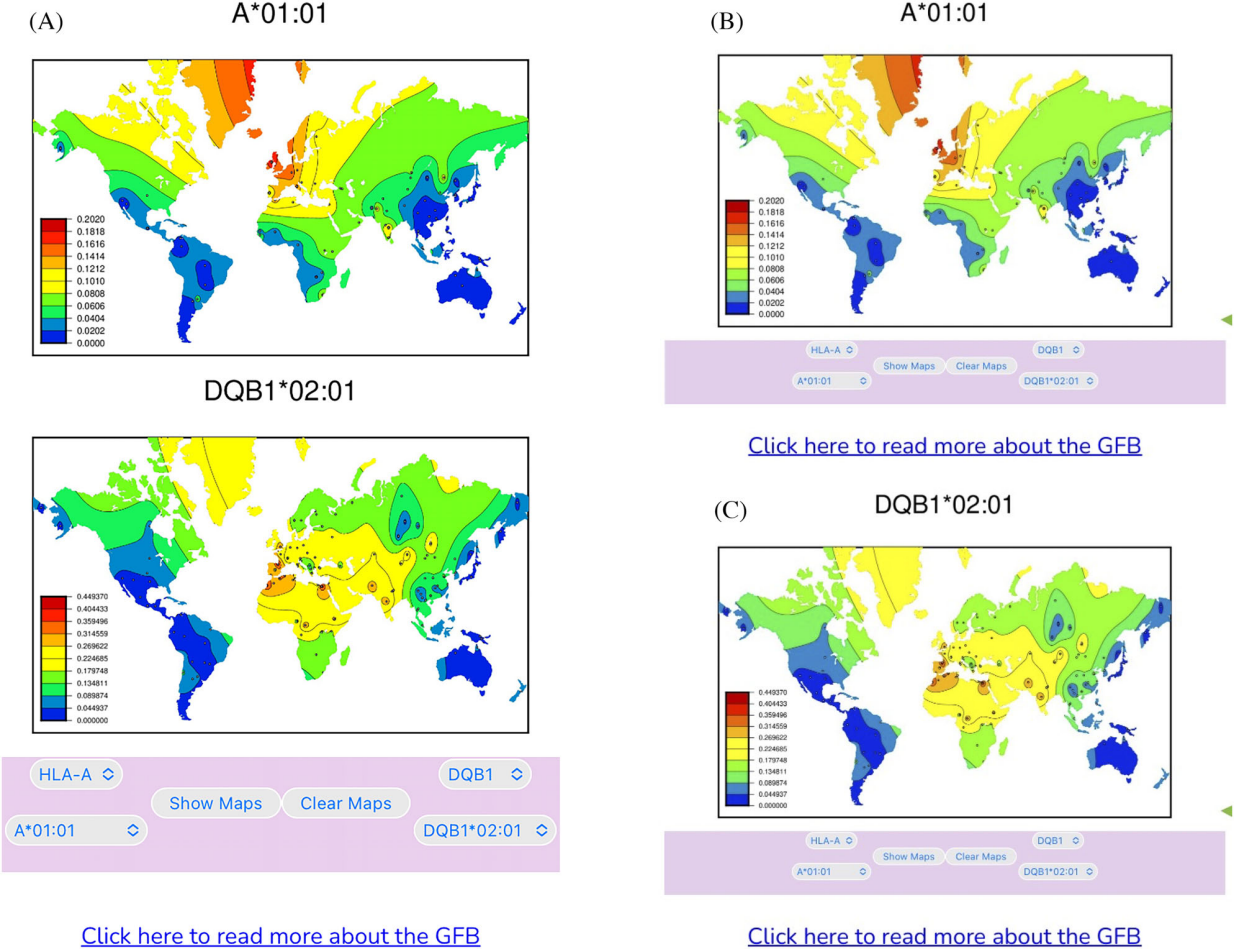
The Global Frequency Browser mobile application. (a) The GFB mobile app in portrait view. (b) The GFB mobile app in left landscape view. (c) The GFB mobile app in right landscape view. The GFB mobile application presents pairs of color frequency heatmaps for comparison in portrait view, with controls corresponding to those in the GFB web application at the bottom of the screen. When the mobile device is turned to the left, the top allele will be shown in landscape mode, as in Figure 7B. The bottom allele is shown in landscape mode when the mobile device is turned to the right, as in Figure 7C. Green triangles on the right of Figures 7B and 7C indicate the bottom of the screen on an Apple iPhone SE smart phone. Swiping up in the GFB mobile application while in landscape mode will reveal the rest of the map and the browser's controls. A link at the bottom of each page leads to additional information about the GFB.

The mobile GFB runs in any contemporary mobile browser and can be installed as an app on iOS devices. Where the web GFB application presents frequency heatmaps side‐by‐side, the mobile GFB presents the two selected maps aligned vertically when the device is in portrait mode, or presents them individually in landscape mode when the device is turned to the left or the right, as illustrated in Figure 7. A map in landscape mode is larger than a map in portrait mode, and maps in either mode can be rescaled pinching or zooming. All of the maps can be browsed by allele name. The mobile GFB can be installed as an application on iOS devices using the ‘Add to Home Screen’ function, as detailed in the ‘Click here to read more about the GFB’ link at the bottom of the browser.

## 4 Discussion

We have developed three new informatic tools that offer a means to accelerate research on the structure and function of HLA genes and alleles. All are freely available and ready for use. HLAtools is an informatic toolkit that makes static resources for HLA genes, alleles and variants computable, and places them alongside new functions and associated resources, with the aim of accelerating research and discovery. The package contains many functions that have not been discussed here; a large subset of HLAtools functions are focused on enabling and performing data analyses, and the conversion of datasets between different data‐analysis formats for use with other tools. The reader is encouraged to review the HLAtools vignette (Supporting Information 1), and explore these functions. Development of the package is ongoing, and updates will continue to be released. Development plans for the package include the incorporation of reference datasets identifying gene and protein elements that contribute to the secondary and tertiary structural elements of HLA proteins, and the application of sequence feature variant type (Karp et al. 2010, Thomson et al. 2010) definitions for data analysis (e.g., via BIGDAWG) and visualization (e.g., via SSHAARP). Feedback and suggestions from the immunogenomics community are welcomed and can be provided at https://github.com/sjmack/HLAtools/issues.

SSHAARP is more narrowly focused on the generation of frequency heatmaps. While we focus on HLA variants here, frequency data for any locus supported in the ANHIG/IMGTHLA GitHub repository can be used to generate colour or greyscale heatmaps. Amino‐acid motif frequency heatmaps can be generated for the *HLA*, *HFE*, *MICA*, *MICB*, *TAP1* and *TAP2* genes, but this capacity could be extended to expressed genes in the ANHIG/IPDKIR and ANHIG/IPDMHC GitHub repositories in the future. More generally, the capacity for SSHAARP to generate heatmaps is limited by the availability of frequency data. A key goal in the ongoing development of SSHAARP is to standardize access to online population frequency data resources like the AFND (Santos et al. 2016). As with HLAtools, community feedback on SSHAARP is welcomed and can be provided at https://github.com/liviatran/SSHAARP_package/issues.

The GFBs have the most limited scope of these applications but are likely the most accessible, as many in the immunogenomics community may not have the time or capacity to build maps. While 22,509 two‐field *HLA‐A*, *HLA‐B*, *HLA‐C*, *HLA‐DRB1*, *HLA‐DQA1*, *HLA‐DQB1*, *HLA‐DPA1* and *HLA‐DPB1* alleles have been described, most have been observed in a small number of individuals, such that a much smaller fraction of alleles are likely to be observed at detectable frequencies in multiple populations. Version 2.0.0 of the common and well‐documented HLA allele catalogue (Mack et al. 2013) (CWD2) includes 1122 two‐field *HLA‐A*, *HLA‐B*, *HLA‐C*, *HLA‐DRB1*, *HLA‐DQA1*, *HLA‐DQB1*, *HLA‐DPA1* and *HLA‐DPB1* alleles, and the common, intermediate and well‐documented (CIWD) HLA allele catalogue (Hurley et al. 2020) includes 3071 two‐field *HLA‐A*, *HLA‐B*, *HLA‐C*, *HLA‐DRB1*, *HLA‐DQB1*, and *HLA‐DPB*1 alleles. These catalogues comprise 5% and 14% of known two‐field HLA alleles, respectively. Seventy‐seven percent of the 805 GFB allele maps describe the frequencies of two‐field CWD2 alleles and represent 60% of two‐field CWD2 alleles. Eighty‐seven percent of the GFB allele maps describe the frequencies of two‐field CIWD alleles (with 70% representing alleles in the common and intermediate categories), and represent 20% of two‐field CIWD alleles.

It is clear that more maps need to be added to the GFBs, but the current GFB maps already represent a significant fraction of the high‐frequency two‐field HLA alleles in the human population. As availability of and access to global HLA allele‐frequency data improve, new maps will be generated and added to the GFBs. As with HLAtools and SSHAARP, community feedback on the GFBs (at github.com/GlobalFrequencyBrowser/GFB/issues), and participation in expanding its collection of maps, is welcomed.

## 5 Conclusions

The large, and ever‐growing number of known HLA alleles can be daunting. Estimates suggest that there may be 28 million HLA alleles in the human population (Klitz et al. 2012, Robinson et al. 2017). Fortunately, the vast majority of these alleles are expected to be very rare (Klitz et al. 2012, Robinson et al. 2017), and it seems likely that most of the high‐frequency HLA alleles in the human population have already been detected (Sanchez‐Mazas and Nunes 2024). Despite this, the size and scope of the IPD‐IMGT/HLA Database will continue to grow, and informatic systems that can process and analyze these data will remain essential for the foreseeable future. HLAtools is designed to accommodate this growth, by making large, difficult to parse HLA datasets computable and challenging HLA undertakings manageable. The frequency heatmaps generated by SSHAARP and made available via the GFBs are currently limited to HLA alleles, but our goal is for SSHAARP to be sufficiently flexible to generate global frequency heatmaps for any locus. GFB stands for ‘Global Frequency Browser’, and not ‘Global HLA Frequency Browser’, precisely because we aim to expand its scope to allow the browsing of frequency data for many other genes. We invite histocompatibility and immunogenetics researchers to use these tools, and encourage the incorporation of these tools into new and existing workflows, as well as novel immunogenetic informatics tools.

## Conflicts of Interest

Livia Tran, Ryan Nickens, Vinh Luu and Effie W. Petersdorf, affirm that they have no conflicts of interest to disclose with respect to the research and work described in this publication, the authorship of this publication or the publication of this article by the International Journal of Immunogenetics. Steven J. Mack is a member of the editorial boards of the *International Journal of Immunogenetics* and the journal *HLA*, and is an Associate Editor for the journal *Human Immunology*. He affirms that he has taken no actions to influence the peer review and/or publication of the work described in this publication and has no financial relationship with the *International Journal of Immunogenetics*, its editor‐in‐chief or its publisher. He affirms that he has no financial conflicts of interests to disclose with respect to the research and work described in this publication, the authorship of this publication or the publication of this article by the International Journal of Immunogenetics.

## Supporting information




**Supporting File 1**: iji70013‐sup‐0001‐SuppMat.pdf

**Supporting File 2**: iji70013‐sup‐0002‐SuppMat.pdf

**Supporting File 3**: Locations of *HLA‐Y* and *HLA‐R* in the Class I Region.
*DRB* Gene Locations on Six Human Genome Reference Assemblies.

**Supporting File 4**: iji70013‐sup‐0005‐figureS1.png

**Supporting File 5**: iji70013‐sup‐0005‐figureS2.png

## Data Availability

The authors have nothing to report.
