# Supplementary material for: HLAtools, Searching Shared HLA Amino Acid Residue Prevalence, and the Global Frequency Browsers: New Computational Resources for Working With HLA Data and Visualizing Global Patterns of HLA Variation
Source: Int J Immunogenet. 2025 Sep 14;52(6):358–70. doi: 10.1111/iji.70013 (PMC12595587; doi:10.1111/iji.70013)
Supplement: Supplementary file 1 — Supporting File 1: iji70013‐sup‐0001‐SuppMat.pdf [file IJI-52-358-s001.pdf]

HLAtools

Ryan Nickens – rynickens@gmail.com

Livia Tran – livia.tran@ucsf.edu

Leamon Crooms IV – lcroomsiv@gmail.com

Derek Pappas, PhD – djpappas75@gmail.com

Vinh Luu – vinhluu864@berkeley.edu

Josh Bredeweg

Steven J. Mack, PhD – steven.mack@ucsf.edu

```
library(HLAtools)
```

- Package Version: 1.6.3

Overview

The *HLAtools* package contains a set of computable resources and a suite of tools intended to facilitate the effective application and analysis of the named genes in the HLA region.

HLAtools includes:

- Reference datasets that describe the genes supported by the IPD-IMGT/HLA Database, organize them into useful categories, and identify their gene feature boundaries.
- The ability to build local amino acid, codon, coding nucleotide, and genomic nucleotide sequence alignments for HLA genes of interest.
- Advanced tools of the comparison and dissection of allele names and allele sequences, and the construction of customized multi-gene sequence alignments.
- Functions that translate allele names, vectors and data frames of allele names, and Genotype List (GL) String Code messages across IPD-IMGT/HLA database release versions, and inter-convert between GL String and UNIFORMAT formats.
- Data-analysis tools that extend the utility of Bridging Immunogenomic Data-Analysis Workflow Gaps (BIGDAWG) formatted datasets by performing multiallele and multilocus stratification, calculating Relative Risk measures, and converting BIGDAWG datasets to PyPop datasets.

---

1. Reference Datasets

Atlas of Gene Feature Boundaries

The *HLAatlas* data object is a list object of sub-lists of R data frames identifying the location of boundary positions between gene-features (exons, introns and untranslated regions [UTRs]) identified in the protein, nucleotide and genomic alignments for each gene supported in the ANHIG/IMGTHLA GitHub Repository. Example protein (*prot*), nucleotide (*nuc*) and genomic (*gen*) atlases for HLA-A are shown below. A new *HLAatlas* can be built after each IPD-IMGT/HLA Database release using the *updateAll()* function, although the atlases are not expected to change unless new genes are added to the IPD-IMGT/HLA Database.

Building the *HLAatlas* object requires internet access, and it can take several minutes to build a full complement of atlases. For example, building all of the atlases for IPD-IMGT/HLA Database release version 3.56.0 took 4.2 minutes on a 3.3 GHz 12-Core Intel Xenon W 2019 Mac Pro with 288 GB of 2933 MHZ DDR4 RAM, 4.6 minutes on a 2.4 GHz 8-Core Intel Core i9 2019 MacBook Pro with 64 GB of 2667 MHZ DDR4 RAM, and 5.24 minutes on a 2.4 GHz Intel Core i9-10885H Dell Precision 5500 with 32 GB of RAM.

HLAatlas objects can be built for all HLA region genes with available genomic, nucleotide and amino acid alignments in release versions 3.00.0 to 3.59.0. Barring changes in the structure of the source data for this object in future IPD-IMGT/HLA Database releases, it should be possible to build *HLAatlas* objects for future releases.

Protein Sequence Atlases

The column headers for *prot* atlases identify the peptide residues encoded by codons that follow or contain an exon (E) boundary. The HLA-A *prot* atlas below illustrates that the codon encoding amino acid 1 included the Exon 1: Exon 2 boundary (E.1-2).

```
HLAatlas$prot$A
   E.1-2 E.2-3 E.3-4 E.4-5 E.5-6 E.6-7 E.7-8
AA     1    91   183   275   314   325   341
```

Nucleotide Sequence Atlases

The column headers for *nuc* atlases identify the cDNA and codon positions preceded by an exon (E) boundary. The HLA-A *nuc* atlas below illustrates that the Exon 1:Exon 2 boundary (E.1-2) is between nucleotide positions 73 and 74 and within codon 1.

```
HLAatlas$nuc$A
      E.1-2 E.2-3 E.3-4 E.4-5 E.5-6 E.6-7 E.7-8
cDNA     74   344   620   896  1013  1046  1094
codon     1    91   183   275   314   325   341
```

Genomic Sequence Atlases

The column headers for *gen* atlases identify the genomic nucleotide positions preceded by a UTR (U), exon (E) or intron (I) boundary. The HLA-A *gen* atlas below illustrates that the boundary between the 5’ UTR (U.5) and Exon 1 (E.1) is between genomic positions -1 and 1, and that the boundary between Intron 3 and Exon 4 is between positions 1569 and 1570 (presented as a table for readability).

	U.5-E.1	E.1-I.1	I.1-E.2	E.2-I.2	I.2-E.3	E.3-I.3	I.3-E.4	E.4-I.4	I.4-E.5	E.5-I.5	I.5-E.6	E.6-I.6	I.6-E.7	E.7-I.7	I.7-E.8	E.8-U.3
gDNA	1	74	204	474	715	991	1570	1846	1948	2065	2507	2540	2682	2730	2899	2904

Historical Catalogue of HLA Allele Names

The *alleleListHistory* data object is a data frame that identifies all HLA, MIC and TAP allele names and their accession identifiers (HLA_IDs) for all IPD-IMGT/HLA Database release versions (e.g., “X3350” represents version 3.35.0) going back to version 1.05.0 (January of 2000), and includes allele names that have been changed or deleted. A new *alleleListHistory* can be built after each IPD-IMGT/HLA Database release using the *updateAll()* function.

```
alleleListHistory$AlleleListHistory[1:5,1:5]
    HLA_ID           X3560           X3550           X3540           X3530
1 HLA00001  A*01:01:01:01  A*01:01:01:01  A*01:01:01:01  A*01:01:01:01
2 HLA00002  A*01:02:01:01  A*01:02:01:01  A*01:02:01:01  A*01:02:01:01
3 HLA00003  A*01:03:01:01  A*01:03:01:01  A*01:03:01:01  A*01:03:01:01
4 HLA00004 A*01:04:01:01N A*01:04:01:01N A*01:04:01:01N A*01:04:01:01N
5 HLA00005  A*02:01:01:01  A*02:01:01:01  A*02:01:01:01  A*02:01:01:01

alleleListHistory$AlleleListHistory[c(65,100:105,2094),c(1,23,58:59,81,96,101
,102)]
       HLA_ID          X3350      X3000     X2280  X2090  X1110  X1060  X1050
65   HLA00069        A*24:19    A*24:19    A*2419 A*2419 A*2419   <NA>   <NA>
100  HLA00107        A*33:04    A*33:04    A*3304 A*3304 A*3304 A*3304 A*3304
101  HLA00108 A*34:01:01:01 A*34:01:01  A*340101 A*3401 A*3401 A*3401 A*3401
102  HLA00109 A*34:02:01:01    A*34:02    A*3402 A*3402 A*3402 A*3402 A*3402
103  HLA00110        A*36:01    A*36:01    A*3601 A*3601 A*3601 A*3601 A*3601
104  HLA00111        A*43:01    A*43:01    A*4301 A*4301 A*4301 A*4301 A*4301
105  HLA00112 A*66:01:01:01    A*66:01    A*6601 A*6601 A*6601 A*6601 A*6601
2094 HLA02186      MICB*021N  MICB*021N MICB*021N   <NA>   <NA>   <NA>   <NA>
```

The *alleleListHistory* data object is expected to change with each IPD-IMGT/HLA Database release. Third-party functions that reference the *alleleListHistory* data object should rely

on an internal version of this data object built using the *HLAtools::updateAlleleListHistory()* function.

Functional and Organizational Categories of Genes in the HLA Region

Molecular Characteristics of HLA Region Genes

The *IMGTHLAGeneTypes* data object describes the named genes within the HLA region curated by the IPD-IMGT/HLA Database. *IMGTHLAGeneTypes* distinguishes pseudogenes and gene fragments from expressed genes, and summarizes each gene's molecular characteristics.

The information in this object can be found online at https://hla.alleles.org/pages/genes/genes_list/. A new *IMGTHLAGeneTypes* can be built using the *updateAll()* function, although the source data is not expected to change unless new genes are added to the IPD-IMGT/HLA Database.

Gazetteer of HLA Region Genes

The *HLAgazetteer* data object is a list object that organizes the HLA region genes supported by the IPD-IMGT/HLA Database in nineteen vectors describing the availability of alignments, gene functionality, group identity and map order. A new *HLAgazetteer* can be built after each IPD-IMGT/HLA Database release using the *updateAll()* function, although the *HLAgazetteer* is not expected to change unless new genes are added to the IPD-IMGT/HLA Database.

```
names(HLAgazetteer)
 [1] "align" "gen" "nuc" "prot" "nogen" "nonuc" "noprot" "pseudo" "frag" "hla
" "expressed" "notexpressed" "classireg" "classihla" "classiireg" "classiihla
" "classical" "nonclassical" "map" "version"
```

For example, the *$align* vector includes all of the genes for which **sequence alignments** are available:

```
HLAgazetteer$align
 [1] "A" "B" "C" "DMA" "DMB" "DOA" "DOB" "DPA1" "DPA2" "DPB1" "DPB2" "DQA1" "
DQA2" "DQB1" "DQB2" "DRA"  "DRB1" "DRB2" "DRB3" "DRB4" "DRB5" "DRB6" "DRB7" "
DRB8" "DRB9" "E" "F" "G" "H" "HFE" "J" "K" "L" "MICA" "MICB" "N" "P" "S" "T"
"TAP1" "TAP2" "U" "V" "W" "Y"
```

The *$prot* vector includes all of the genes with **protein** alignments:

```
HLAgazetteer$prot
 [1] "A" "B" "C" "DMA" "DMB" "DOA" "DOB" "DPA1" "DPB1" "DQA1" "DQA2" "DQB1" "
DQB2" "DRA" "DRB1" "DRB3" "DRB4" "DRB5" "DRB" "E" "F" "G" "HFE" "MICA" "MICB"
"TAP1" "TAP2"
```

Note that the *$prot* and *$nuc* vectors include a 'DRB' "gene". While 'DRB' is not a gene name, the DRB_prot.txt file includes combined alignments for the DRB1, DRB3, DRB4, and

DRB5 genes, and the DRB_nuc.txt file includes combined alignments for the DRB1, DRB2, DRB3, DRB4, DRB5, DRB6, DRB7, DRB8, and DRB9 genes. 'DRB' is included in these vectors for the purpose of validation when these combined alignments are desired.

Annotation of Pseudogene and Gene Fragment Features

The *fragmentFeatureNames* object identifies and annotates the non-standard gene features found in pseudogenes and gene fragments, based on the positions of feature boundaries ("|") in the pertinent genomic alignment sequence. Where the features of functional genes are limited to Introns (I), Exons (E) and Untranslated Regions (U), the non-standard features in pseudogenes and gene fragments are described as:

- Joins (J), when a feature includes sequence from two or more sequences that are separated by a boundary in the reference
- Segments (S), when the sequence of a feature is a subset of a longer feature sequence
- Novel (N), when the sequence of a feature does not correspond to a known sequence
- Hybrid (H), when a feature includes a sequence to belongs to a known feature as well as nucleotide sequence that does not correspond to a feature

For each pseudogene or gene fragment, the *fragmentFeatureNames* element contains a $features element identifying the gene features in the 5' to 3' direction, and an $annotation element that provides some detail about the feature.

These non-standard gene features are included in the feature boundaries of the gene fragments and pseudogenes in the *HLAatlas*.

The current annotations should not change across IPD-IMGT/HLA Database releases. When a new pseudogene or gene-fragment is added to the IPD-IMGT/HLA Database, a new annotation will be added as part of a package update.

```
fragmentFeatureNames$DPA2
$features
[1] "U.5" "E.1" "I.1" "E.2" "I.2" "E.3" "I.3" "E.4" "U.3"
$annotation
[1] "All of the reference gene features are present. E.1 starts 28 nucleotide
s before the reference, and ends 88 nucleotides before the reference."

fragmentFeatureNames$L
$features
 [1] "U.5" "E.1" "I.1" "E.2" "I.2" "E.3" "I.3" "E.4" "I.4" "E.5" "I.5" "E.6"
"I.6" "E.7" "I.7" "E.8" "U.3"
$annotation
[1] "All of the reference gene features are present."

fragmentFeatureNames$P
```

```
$features
 [1] "J.1" "E.3" "I.3" "E.4" "I.4" "E.5" "I.5" "E.6" "I.6" "E.7" "I.7" "U.3"
$annotation
[1] "J.1 is ~350 nucleotides of novel sequence followed by ~120 nucleotides f
rom the 5' end of Intron 2. Exon 8 is absent."

fragmentFeatureNames$S
$features
[1] "H.1" "S.1" "J.1" "E.7" "I.7" "E.9" "S.2"
$annotation
[1] "H.1 is 37 nucleotides of novel sequence, followed by the last 185 nucleo
tides of Intron 5. S.1 is the first 27 nucleotides of Exon 6. J.1 is the last
4 nucleotides of Exon 6 (2 nucleotides in the reference have been deleted), f
ollowed by the last 100 nucleotides of Intron 6 (6 nucleotides in the referen
ce have been deleted). E.9 is a 191 nucleotide-long Exon in what is the 5' en
d of the 3' UTR in the reference. S.2 is the 3' end of the 3' UTR."
```

Sequence Alignments

The *HLAalignments* data object is a list object of sub-lists of data frames of peptide (*prot*), codon (*codon*), coding nucleotide (*nuc*) and genomic nucleotide (*gen*) alignments for the HLA and HLA-region genes supported in the ANHIG/IMGTHLA GitHub Repository, along with a *version* character string that identifies the IPD-IMGT/HLA Database release under which the *HLAalignments* object was built.

Given the size of all the combined alignments, *HLAalignments* **is not bundled with the HLAtools package**. All alignments, or desired subsets of each type of alignment for a given gene, can be built using the *alignmentFull()* function, as described in Section 2 *Working with Sequence Alignments*, below. *HLAalignments* objects can be built for all HLA region genes with available genomic, nucleotide and amino acid alignments in IPD-IMGT/HLA release versions 3.00.0 to 3.59.0. Barring changes in the structure of the source data for this object in future IPD-IMGT/HLA Database releases, it should be possible to build *HLAalignments* objects for future releases.

These protein, codon, nucleotide and genomic DNA sequence alignments are described in detail below.

The first four columns of each alignment identify the locus, allele, two-field allele name (trimmed_allele), and full allele name (allele_name) for each sequence, while the remaining columns identify the sequence at each position, as illustrated below.

Protein (AA) Aligments

The column header for the *prot* alignments identifies individual peptide positions, starting from the first peptide of the leader sequence (a negative position) or the first peptide of the native protein when there is no leader sequence. For example, as shown below, the HLA-A *prot* alignment begins at residue -24.

```
HLAalignments$prot$A[1:5,1:15]
  locus       allele trimmed_allele    allele_name -24 -23 -22 -21 -20 -19 -1
8 -17 -16 -15 -14
1     A  01:01:01:01        A*01:01  A*01:01:01:01   M   A   V   M   A   P
R   T   L   L   L
2     A 01:01:01:02N        A*01:01N A*01:01:01:02N   M   A   V   M   A   P
R   T   L   L   L
3     A  01:01:01:03        A*01:01  A*01:01:01:03   M   A   V   M   A   P
R   T   L   L   L
4     A  01:01:01:04        A*01:01  A*01:01:01:04   M   A   V   M   A   P
R   T   L   L   L
5     A  01:01:01:05        A*01:01  A*01:01:01:05   M   A   V   M   A   P
R   T   L   L   L
```

In contrast, the TAP *prot* alignment begins at residue 1, as shown below.

```
HLAalignments$prot$TAP1[1:5,1:15]
  locus      allele trimmed_allele      allele_name 1 2 3 4 5 6 7 8 9 10 11
1  TAP1 01:01:01:01     TAP1*01:01 TAP1*01:01:01:01 M A S S R C P A P  R  G
2  TAP1 01:01:01:02     TAP1*01:01 TAP1*01:01:01:02 M A S S R C P A P  R  G
3  TAP1 01:01:01:03     TAP1*01:01 TAP1*01:01:01:03 M A S S R C P A P  R  G
4  TAP1 01:01:01:04     TAP1*01:01 TAP1*01:01:01:04 M A S S R C P A P  R  G
5  TAP1 01:01:01:05     TAP1*01:01 TAP1*01:01:01:05 M A S S R C P A P  R  G
```

Codon and Individual Nucleotide (cDNA) Sequence Alignments

Codon-triplet (*codon*) and individual nucleotide (*nuc*) alignments are included as separate data frames.

The column heads for the *codon* alignments identify the individual nucleotide positions within each codon, starting with the first codon. As shown below, the HLA-A *codon* alignment begins at the first nucleotide in codon position -24, which is identified as *-24*; the second nucleotide in codon -24 is identified as *-24.1* and the third nucleotide in codon -24 is identified as *-24.2*.

```
HLAalignments$codon$A[1:5,1:12]
  locus       allele trimmed_allele    allele_name -24 -24.1 -24.2 -23 -23.1
-23.2 -22 -22.1
1     A  01:01:01:01        A*01:01  A*01:01:01:01   A     T     G   G     C
C   G     T
2     A 01:01:01:02N        A*01:01N A*01:01:01:02N   A     T     G   G     C
C   G     T
3     A  01:01:01:03        A*01:01  A*01:01:01:03   A     T     G   G     C
C   G     T
4     A  01:01:01:04        A*01:01  A*01:01:01:04   A     T     G   G     C
C   G     T
5     A  01:01:01:05        A*01:01  A*01:01:01:05   A     T     G   G     C
C   G     T
```

The column heads for the *nuc* alignments identify each individual nucleotide position, starting from the first transcribed nucleotide (1), as shown below.

```
HLAalignments$nuc$A[1:5,1:21]
  locus       allele trimmed_allele    allele_name 1 2 3 4 5 6 7 8 9 10 11 12
13 14 15 16 17
1     A  01:01:01:01        A*01:01  A*01:01:01:01 A T G G C C G T C  A  T  G
G  C  G  C  C
2     A 01:01:01:02N        A*01:01N A*01:01:01:02N A T G G C C G T C  A  T
G  G  C  G  C  C
3     A  01:01:01:03        A*01:01  A*01:01:01:03 A T G G C C G T C  A  T  G
G  C  G  C  C
4     A  01:01:01:04        A*01:01  A*01:01:01:04 A T G G C C G T C  A  T  G
G  C  G  C  C
5     A  01:01:01:05        A*01:01  A*01:01:01:05 A T G G C C G T C  A  T  G
G  C  G  C  C
```

Genomic (gDNA) Sequence Alignments

The column heads for the *gen* alignments identify the individual nucleotide positions in the 'full gene' sequence, which usually starts in the 5' untranslated region (UTR) sequence for expressed genes.

```
HLAalignments$gen$A[1:5,1:13]
  locus       allele trimmed_allele    allele_name -300 -299 -298 -297 -296 -
295 -294 -293 -292
1     A  01:01:01:01        A*01:01  A*01:01:01:01    C    A    G    G    A
G    C    A    G
2     A 01:01:01:02N        A*01:01N A*01:01:01:02N    *    *    *    *    *
*    *    *    *
3     A  01:01:01:03        A*01:01  A*01:01:01:03    C    A    G    G    A
G    C    A    G
4     A  01:01:01:04        A*01:01  A*01:01:01:04    *    *    *    *    *
*    *    *    *
5     A  01:01:01:05        A*01:01  A*01:01:01:05    *    *    *    *    *
*    *    *    *
```

Insertion-Deletion (Indel) Variant Representation

Indel positions in these alignments are numbered sequentially, following the first position 5'/N-terminal of the indel, with decimal values appended to the 5' reference position in the indel positions, starting from ".1", as illustrated below.

```
HLAalignments$nuc$DQB1[c(1,1138:1140),c(1:4,57:63)]
     locus      allele trimmed_allele      allele_name 53 54 54.1 54.2 54.3 5
4.4 55
1     DQB1 05:01:01:01     DQB1*05:01 DQB1*05:01:01:01  T  C    .    .    .
.  A
1138  DQB1      06:421    DQB1*06:421      DQB1*06:421  *  *    .    .    .
.  *
```

```
1139  DQB1     06:422N   DQB1*06:422N     DQB1*06:422N  T  C    T    G    T
C  A
1140  DQB1     06:423N   DQB1*06:423N     DQB1*06:423N  *  *    .    .    .
.  *

HLAalignments$gen$DQB1[c(1,378:380),c(1:4,589:594)]
    locus      allele trimmed_allele      allele_name 52 52.1 52.2 52.3 52.4
53
1    DQB1 05:01:01:01     DQB1*05:01 DQB1*05:01:01:01  G    .    .    .    .
T
378  DQB1 06:41:01:03     DQB1*06:41 DQB1*06:41:01:03  G    .    .    .    .
T
379  DQB1     06:422N   DQB1*06:422N     DQB1*06:422N  G    T    C    T    G
T
380  DQB1      06:424    DQB1*06:424      DQB1*06:424  G    .    .    .    .
T
```

Indels in *codon* alignment positions are similarly numbered from the codon in the N-terminal direction. However, due to a peculiarity of the R environment, which generates unique column-header names, the last two positions of the N-terminal-ward codon **before** the deletion will be numbered with the decimal values following those used to identify the insertion positions, as shown below. This only occurs when indel positions are included in a selected range. In this example, the *allele* and *trimmed_allele* columns have been omitted for spacing.

```
HLAalignments$codon$DQB1[c(1,1138:1140),c(1,4,56:65)]
     locus      allele_name -15 -15.5 -15.6 -15.1 -15.2 -15.3 -15.4 -14 -14.1
-14.2
1     DQB1 DQB1*05:01:01:01   G     T     C     .     .     .     .   A     C
C
1138  DQB1      DQB1*06:421   *     *     *     .     .     .     .   *     *
*
1139  DQB1     DQB1*06:422N   G     T     C     T     G     T     C   A     C
C
1140  DQB1     DQB1*06:423N   *     *     *     .     .     .     .   *     *
*
```

---

2. Working with Sequence Alignments

As noted above, the **HLAtools** package includes several functions for working with sequence alignments. All of these functions require that alignments first be built, as the complete set of protein, codon, nucleotide and genomic sequence alignments is too large to include in the **HLAtools** package. **The functions that perform operations in sequence alignments expect the alignments to be in a variable named *HLAalignments***. For example the HLA-A protein alignment is identified as *HLAalignments$prot$A*. If alignments

are built into a different data object, they will not be accessible to the functions described below.

Building Alignments

alignmentFull() is a wrapper function that applies *buildAlignments()* to populate the *HLAalignments* object. When *alignmentFull()* is run, the resulting alignments will be built using the IPD-IMGT/HLA Database release under which the *HLAgazetteer* object was built ('HLAgazetteer$version'). When alignments for a different database release are desired, *updateAll()* should be used to update the *HLAgazetteer* to the desired release version before applying *alignmentFull()*.

Requirements

The *alignmentFull()* function requires internet access, and can take significant time to run to build a full complement of alignments.

For example, to build all of the alignments in IPD-IMGT/HLA Database release version 3.56.0, *HLAalignments* <- *alignmentFull()* completed in 6.2 minutes on a 3.3 GHz 12-Core Intel Xenon W 2019 Mac Pro with 288 GB of 2933 MHZ DDR4 RAM, 6.5 minutes on a 2.4 GHz 8-Core Intel Core i9 2019 MacBook Pro with 64 GB of 2667 MHZ DDR4 RAM, and 9.9 minutes on a 2.4 GHz Intel Core i9-10885H Dell Precision 5500 with 32 GB of RAM.

Parameters

- *loci*: A vector of the locus names for which alignments should be built. The default value ("all") specifies all loci. Allowed loci are listed in *HLAgazetteer$align*.
- *alignType*: A vector of alignment types for which alignments should be built. Allowed values are "prot", "codon", "nuc" and "gen", which specify respective protein, codon, nucleotide and genomic alignments, as well as the default value ("all"), which specifies all four alignment types. Loci with alignments for a specific *alignType* are defined in *HLAgazetteer$prot*, *HLAgazetteer$nuc* and *HLAgazetteer$gen*.
- *version*: A character string identifying the IPD-IMGT/HLA Database release version for which alignments should be built. The default value ("Latest") generates alignments for the most recent IPD-IMGT/HLA Database release. Other releases are identified by their IPD-IMGT/HLA Database version; e.g., "3.55.0" identifies IPD-IMGT/HLA Database version 3.55.0.

Return A list object containing data frames of protein (*prot*), codon (*codon*), coding nucleotide (*nuc*), and genomic nucleotide (*gen*) alignments for specified genes in the specified IPD-IMGT/HLA Database release, and a character string identifying the pertinent reference database version is returned.

Generate a full set of all alignments for all supported genes in the current release.

```
HLAalignments <- alignmentFull()
```

Generate all alignments for four genes in the current release.

```
HLAalignments <- alignmentFull(loci = c("C","DQB1","DPA1","DRB5"))
```

Generate a protein alignment for one gene in release version 3.54.0.

```
updateAll(updateType = "HLAgazetteer",version = "3.54.0")
HLAalignments <- alignmentFull("DRB1","prot","3.54.0")
```

Working with Alignments in Past Releases

As indicated above, *alignmentFull()* can be applied for any IPD-IMGT/HLA Database release version. The *updateAll()* function must be applied to build the *HLAgazetteer* for the desired release version **before** applying *alignmentFull()*.

```
> updateAll()
IMGTHLAGeneTypes for version 12-10-2022 is already loaded.
HLAgazetteer for version 3.56.0 has been built and loaded.
alleleListHistory for version 3.56.0 has been built and loaded
fragmentFeatureNames for version 3.56.0 has been built and loaded.
HLAatlas for version 3.56.0 has been built and loaded.

HLAalignments <- alignmentFull()
```

Caveats for Alignments from Previous Releases

Prior to IPD-IMGT/HLA Database release version 3.24.0, the names of the sequence alignment files for the HLA-DP and HLA-DQ genes in the IPD-IMGT/HLA GitHub Repository did not include a numerical suffix in the gene name (e.g., the protein sequence alignment file for the DQA1 gene was 'DQA_prot.txt') because sequence alignment files for the DPA2, DPB2, DQA2 and DQB2 genes had not been made available.

Similarly, prior to release version 3.51.0, the 'DRB_prot.txt' and 'DRB_nuc.txt' alignment files were the source of alignments for DRB1, DRB3, DRB4 and DRB5. In release versions 3.0.0 to 3.23.0, genomic DRB1 alignments are in 'DRB_gen.txt' files, and these alignments are in 'DRB1_gen.txt' files in subsequent releases. Building DPA1, DPB1, DQA1, DQB1, DRB1, DRB3, DRB4 and DRB5 sequence alignments from these earlier releases may require specifying a gene name that does not include the numerical suffix. When the *HLAgazetteer* has been updated to the desired release version, the available gene names for that release can be found in the *HLAgazetteer*.

In all version 3.*.* releases (3.0.0 to 3.59.0), nucleotide alignments for the DRB2, DRB6, DRB7 and DRB9 genes are included in the 'DRB_nuc.txt' file.

As noted above, when building alignments for past releases, the *HLAgazetteer* should be updated to reflect the gene names included in that release, and consulted to determine which gene names should be provided to alignmentFull().

```
updateAll(version = "3.20.0")
IMGTHLAGeneTypes for version 12-10-2022 is already loaded.
HLAgazetteer for version 3.20.0 has been built and loaded.
alleleListHistory for version 3.20.0 has been built and loaded
fragmentFeatureNames for version 3.20.0 has been built and loaded.
HLAatlas for version 3.20.0 has been built and loaded.

HLAgazetteer$version
[1] "3.20.0"
HLAgazetteer$align
 [1] "A" "B" "C" "DMA" "DMB" "DOA" "DOB" "DPA" "DPB" "DQA" "DQB" "DRA" "DRB3"
"DRB4" "DRB" "E" "F" "G" "H" "J" "K" "L" "MICA" "MICB" "P" "TAP1" "TAP2" "V"
"Y"

HLAalignments <- alignmentFull(c("C","DQB"),alignType = c("nuc","gen"),versio
n = "3.20.0")
```

---

3. Trim, Search and Query Functions

Trimming HLA Allele Names by Digits or Fields

The *multiAlleleTrim()* function shortens HLA allele names in a vector to a specified number of fields or digits, depending on the pertinent nomenclature epoch. Epoch 1 allele names are found in IPD-IMGT/HLA Database releases 1.0.0 to 1.16.0, epoch 2 allele names are found in releases 2.0.0 to 2.28.0, and epoch 3 allele names are found in releases 3.0.0 and onward. Expression variant suffixes in full-length allele names can be appended to truncated allele names, but will not be removed from full-length allele names that have fewer than four fields or eight digits.

The vector must contain only allele names, and all allele names in the vector must belong to the same nomenclature epoch.

- *alleles*: A vector of HLA allele names from a single nomenclature epoch.
- *resolution*: A number identifying the number of fields to which the alleles should be trimmed.
- *version*: A number identifying the HLA nomenclature epoch under which the allele was named. The default value is 3.
- *append*: A logical. When append = TRUE, the expression variant suffix of a full-length allele name is appended to a truncated allele name. The default value is FALSE.

Return A vector of trimmed allele names, shortened according to the input parameters, is returned.

```
multiAlleleTrim(alleles = "A*03:01:01", resolution = 2)
[1] "A*03:01"

multiAlleleTrim(alleles = "A*0303N",resolution = 1,version = 1, append = TRUE
)
[1] "A*03N

multiAlleleTrim("HLA-A*24020102L",resolution = 3,version = 2, append = TRUE)
[1] "HLA-A*240201L"

alleles <- c("A*02:01:01:02L","DRB1*08:07", "DQB1*04:02:01:16Q")
multiAlleleTrim(alleles,2)
[1] "A*02:01"    "DRB1*08:07" "DQB1*04:02"

multiAlleleTrim(alleles,2,append = TRUE)
[1] "A*02:01L"    "DRB1*08:07"  "DQB1*04:02Q"
```

Identifying Differences Between Alleles at a Locus

The *compareSequences()* function identifies sequence differences between two alleles at a locus for a specific type of alignment.

Parameters

- *alignType*: the alignment type of either “codon”, “gen”, nuc” or “prot”.
- *alleles*: a vector of the two alleles to be compared, e.g., “c(”DPA1*01:03:38:01”,“DPA1*01:03:38:02”)“. The ‘HLA-’ prefix is excluded for HLA alleles.

Return If there are no differences between the alleles for the specified ‘alignType’, a message is returned. When there are differences, a data frame identifying the positions and sequences variants that distinguish the alleles is returned.

```
compareSequences("prot",c("DPA1*01:03:38:01","DPA1*01:03:38:02"))
[1] "There are no differences between DPA1*01:03:38:01 and DPA1*01:03:38:02 i
n the protein alignment."

compareSequences("nuc",c("DPA1*01:03:38:01","DPA1*01:03:38:02"))
[1] "There are no differences between DPA1*01:03:38:01 and DPA1*01:03:38:02 i
n the nucleotide alignment."

compareSequences("codon",c("DPA1*01:03:38:01","DPA1*01:03:38:02"))
[1] "There are no differences between DPA1*01:03:38:01 and DPA1*01:03:38:02 i
n the codon alignment."

compareSequences("gen",c("DPA1*01:03:38:01","DPA1*01:03:38:02"))
       allele_name 1544 1723 3318 4149
```

```
1 DPA1*01:03:38:01    G    G    C    G
2 DPA1*01:03:38:02    A    A    G    C
```

Searching Allele Names Across IPD-IMGT/HLA Database Release Versions

The *multiQueryRelease()* function searches the AlleleListHistory object for user-defined allele name variants in a specific IPD-IMGT/HLA release.

Parameters

- *rel*: An IPD-IMGT/HLA Database release version, represented as either a character (e.g. “3.56.0”) or a numeric (e.g., 3560) value.
- *variants*: A vector of character strings representing any part of a locus or allele name (e.g., “DR”, “02:01”, “DRB1*08:07”). The default (““) specifies all alleles in ‘rel’.
- *all*: A logical. When ‘all’ = TRUE, a vector of all instances of ‘variants’ in ‘rel’ is returned. When ‘all’ = FALSE, the number of instances of ‘variants’ in ‘rel’ is returned.

```
multiQueryRelease("3.30.0","DRB9",FALSE)
[1] 1
multiQueryRelease("3.30.0","DRB9",TRUE)
[1] "DRB9*01:01"
multiQueryRelease("3.31.0","DRB9",FALSE)
[1] 6
multiQueryRelease("1.05.0","304",TRUE)
[1] "A*0304"    "A*3304"    "B*1304"    "Cw*03041"  "Cw*03042"  "DQB1*0304" "
DRB1*0304" "DRB1*1304" "B*5304"    "A*2304"
multiQueryRelease("3.58.0",c("DPB","2N"),TRUE)
[1] "DPB1*786:01:02N" "DPB1*401:01:02N"
```

The *verifyAllele()* function searches the AlleleListHistory object to determine if a specific allele name ever existed, and optionally returns the IPD-IMGT/HLA Database release versions in which that allele name existed and the allele’s accession number.

```
verifyAllele("A*02011",TRUE)
[1] "TRUE"   "1.15.0"
> verifyAllele("A*02011",TRUE,TRUE)
[1] "TRUE"   "1.15.0" "1.14.0" "1.13.0" "1.12.0" "1.11.0" "1.10.0" "1.09.0" "
1.08.0" "1.07.0" "1.06.0" "1.05.0"
> verifyAllele("A*02011",TRUE,TRUE,TRUE,TRUE)
[1] "TRUE"     "1.05.0"   "HLA00005"
> verifyAllele("A*02011",TRUE,TRUE,FALSE,TRUE)
[1] "TRUE"     "1.15.0"   "1.14.0"   "1.13.0"   "1.12.0"   "1.11.0"   "1.10.0
"   "1.09.0"   "1.08.0"   "1.07.0"   "1.06.0"   "1.05.0"   "HLA00005"
> verifyAllele("A*0201101",TRUE,TRUE,FALSE,TRUE)
[1] FALSE
verifyAllele("A*020101",TRUE,FALSE,TRUE)
```

```
[1] "TRUE"   "1.16.0"
verifyAllele("A*02:01:01:01",TRUE,FALSE,TRUE,TRUE)
[1] "TRUE"     "3.00.0"   "HLA00005"
verifyAllele("A*02:01:01:01",TRUE,FALSE,FALSE,TRUE)
[1] "TRUE"     "3.60.0"   "HLA00005"
```

Searching Alignments at Specific Positions

The *alignmentSearch()* function returns the variants for specific positions in a single allele for a specific type of alignment.

Parameters

- *alignType*: The alignment type of either “codon”, “gen”, nuc” or “prot”.
- *allelename*: A full-length or two-field allele name, including the locus name, but excluding the “-HLA” prefix for HLA alleles.
- *positions*: A vector of sequence positions (e.g., c(1,2,3,4,5), c(1:5), c(1,2,3,15:20,30)). If positions do not exist in an alignment, a message is returned.
- *prefix*: A logical that indicates if the position numbers should be included in the result (TRUE) or excluded (FALSE).
- *sep*: The value used to separate the sequences for each position. If sep=““, a sequence block is returned.

```
alignmentSearch("nuc","DPA1*01:06:03",107)
[1] "107A"
alignmentSearch("nuc","DPA1*01:06:03",c(3,30,130,230))
[1] "3*~30*~130G~230A"
alignmentSearch("nuc","DPA1*01:06:03",c(120,125,126:128,130))
[1] "120T~125C~126G~127T~128T~130G"
alignmentSearch("nuc","DPA1*01:06:03",c(120,125,126:128,130),prefix=FALSE)
[1] "T~C~G~T~T~G"
alignmentSearch("nuc","DPA1*01:06:03",c(120,125,126:128,130),prefix=FALSE,sep
="")
[1] "TCGTTG"
alignmentSearch("nuc","DPA1*01:06:03",c(120,125,126:128,130),prefix=FALSE,sep
=":")
[1] "T:C:G:T:T:G"
```

When sequences include indel-positions, all of the values in the vector of sequence positions must be characters.

```
alignmentSearch("nuc","DPA1*01:103",c(181:182),prefix=FALSE,sep="")
[1] "GA"
alignmentSearch("nuc","DPA1*01:103",c("181","181.1","181.2","182"),prefix=FAL
SE,sep="")
[1] "G..A"
```

Identifying Sequence Variants at Specific Positions and their Frequencies Across all Alleles

The *queryPositions()* function identifies all of the variants at a single position in a specified locus for a specific type of alignment.

Parameters

- *alignType*: The alignment type of either "codon", "gen", nuc" or "prot". Only a single alignType can be specified.
- *locus*: A locus in HLAalignments.
- *positions*: A vector of sequence positions (e.g., 86, c(1,2,3,4,5), c(1:5), c(1,2,3,15:20,30)). If positions do not exist in a specified alignment, a message is returned.
- *table*: A logical value indicating if a data frame describing the counts and frequencies for each variant should be returned (TRUE) or if a vector of all variants should be returned (FALSE). The default value is FALSE.

```
queryPositions("prot","DRB1",86)
 [1] "." "A" "C" "D" "G" "L" "M" "Q" "R" "S" "V" "W" "X"

queryPositions("prot","DRB1",86,TRUE)
$DRB1_86
   Variant Count    Frequency
1        .    71 0.0185620915
2        A     6 0.0015686275
3        C    10 0.0026143791
4        D     5 0.0013071895
5        G  1887 0.4933333333
6        L     4 0.0010457516
7        M     4 0.0010457516
8        Q     1 0.0002614379
9        R     1 0.0002614379
10       S     2 0.0005228758
11       V  1825 0.4771241830
12       W     5 0.0013071895
13       X     4 0.0010457516
```

Searching Alignments for Sequence Motifs

The *motifMatch()* function returns the names of alleles that share specific nucleotide or peptide motifs.

Parameters

- *motif*: A nucleotide or peptide sequence variant motif structured as Locus*#$~#$~#$, where # identifies a variant position, and $ identifies the sequence variant. Any number of variants can be specified.

- *alignType*: The alignment type of either “codon”, “gen”, nuc” or “prot”. Only a single alignType can be specified.
- *full*: A logical value indicating if full (TRUE) or truncated (FALSE) allele names should be returned.

```
motifMatch("A*-21M~2P","prot")
[1] "A*02:774"  "A*11:284"  "A*11:417N" "A*68:216N"

motifMatch("A*196G~301A~3046T","gen",FALSE)
[1] "A*01:09"

motifMatch("A*196G~301A~3046T","gen",TRUE)
[1] "A*01:09:01:01" "A*01:09:01:02"
```

Building Custom Alignments

The *customAlign()* function returns a customized peptide, codon, coding nucleotide or genomic nucleotide alignment for specified alleles at specified positions.

Parameters

- *alignType* The alignment type of either “codon”, “gen”, nuc” or “prot”.
- *alleles* A vector of allele names, which can incude alleles at multiple loci. The ‘HLA-’ prefix is excluded for HLA alleles.
- *positions* Either a single vector positions, against which all loci will be aligned, or a list of vectors of positions, for which there is one vector for each allele in ‘alleles’. Each allele in ‘alleles’ will be aligned on the basis of the corresponding vector.

```
customAlign("codon",c("DPB1*01:01:01:01","DQA1*01:01:01:01","DQB1*05:01:01:01
"),c(1:4,6:9))
            Allele   1   2   3   4   6   7   8   9
1 DPB1*01:01:01:01 AGG GCC ACT CCA AAT TAC GTG TAC
2 DQA1*01:01:01:01 GAA GAC ATT GTG GAC CAC GTT GCC
3 DQB1*05:01:01:01 AGA GAC TCT CCC GAT TTC GTG TAC
```

When the length of vectors in a ‘positions’ list varies, it is recommended to coordinate the values in ‘alleles’ and ‘positions’ so that the longest vector is generated first.

```
customAlign("codon",c("DPB1*01:01:01:01","DQA1*01:01:01:01","DQB1*05:01:01:01
"),list(19:35,1:4,6:9))
              DPB1  19  20  21  22  23  24  25  26  27  28  29  30  31  32  3
3  34  35
1 DPB1*01:01:01:01 AAT GGG ACA CAG CGC TTC CTG GAG AGA TAC ATC TAC AAC CGG GA
G GAG TAC
2             DQA1   1   2   3   4
3 DQA1*01:01:01:01 GAA GAC ATT GTG
4             DQB1   6   7   8   9
5 DQB1*05:01:01:01 GAT TTC GTG TAC
```

4. Data Translation and Conversion Tools

Working with Genotype List String (GL String), GL String Code (GLSC), and UNIFORMAT Data

Genotype List (GL) String is a grammar for describing the relationships between allele names reported in a genotype, using a set of six case-defined "AND" and "OR" delimiters ('?', '^', '|', '+', '~', '~', and '/').

UNIFORMAT is a parallel grammar that uses an alternate set of operators (",", "|", " ", and"~") to describe the relationships between allele names in a genotype. Additional details of UNIFORMAT can be found at hla-net.eu.

GL String Code (GLSC) is a format that encapsulates a GL String with information describing the associated gene family namespace and the nomenclature version (or date) under which the GL String was generated. Additional information about GL String and GL String Code can be found at glstring.org.

An example GLSC for an HLA GL String generated under IPD-IMGT/HLA Database release version 3.01.0 is presented below.

```
hla#3.1.0#HLA-A*02:01~HLA-C*07:02~HLA-B*07:02~HLA-DRB1*15:01~HLA-DQB1*06:02~H
LA-DPB1*04:02+HLA-A*01:01~HLA-C*06:02~HLA-B*57:01~HLA-DRB1*07:01~HLA-DQB1*03:
03~HLA-DPB1*04:01"
```

Translate GL String Codes Across IPD-IMGT/HLA Database Release Versions

updateGL

The *updateGL()* function translates the HLA allele names in a GL String Code across IPD-IMGT/HLA Database release versions, going back to version 1.05.0. Allele names can be matched across release versions via the allele Accession ID, and via allele name fields shared across alleles in a given release, as illustrated below.

Parameters

- *GLStringCode* A string of HLA allele names and operators in GL String Code format, signifying their relation with one another and the associated IMGT/HLA Database release version.
- *Version* A string identifying of the desired IPD-IMGT/HLA Database release version to which the alleles should be updated.
- *expand* A logical that indicates whether user would like to return all allele names that match the allele name in 'GLStringCode' (TRUE), or if only the direct HLA accession ID match should be returned (FALSE).

- *verbose* A logical that indicates if messages regarding the update process should be sent to the console (TRUE) or not (FALSE). The default value is FALSE.

In the examples below, a GLSC containing a single DPB1 allele name, as recorded in release version 1.05.0, is translated to version 3.52.0. In the first example, 'expand = FALSE', and the HLA accession ID of the submitted allele is used to perform the translation to a single allele. In the second example, 'expand = TRUE' and all alleles that match the submitted allele in release 3.52.0 are returned.

```
updateGL("hla#1.05.0#HLA-DPA1*0106", "3.52.0", expand = FALSE)
[1] "hla#3.52.0#HLA-DPA1*01:06:01"

updateGL("hla#1.05.0#HLA-DPA1*0106", "3.52.0", expand = TRUE)
[1] "hla#3.52.0#HLA-DPA1*01:06:01/HLA-DPA1*01:06:02/HLA-DPA1*01:06:03"
```

multiUpdateGL

The *multiUpdateGL()* function translates columns of GL String Code data across IPD-IMGT/HLA Database release versions in data frames. If a single column of a data frame is provided, it must contain GL string codes. If multiple columns are entered, the first column should contain identifiers, which will not be updated. Translations can be performed for all IPD-IMGT/HLA Database release versions in the loaded *alleleListHistory* data object, going back to IPD-IMGT/HLA Database version 1.05.0.

Parameters

- *GLstringArray* An array of HLA allele names and operators in GL String code format identifying their relation with one another and the pertinent IPD-IMGT/HLA Database release version.
- *Version* A text string identifying the desired version to which for the alleles should be updated.
- *expand* A logical specifying whether to include only the direct HLA ID match (FALSE), or all possible allele matches (TRUE). The default value is FALSE.
- *verbose* A logical that indicates if messages regarding the update process should be sent to the console (TRUE) or not (FALSE). The default value is FALSE.

```
GLstringArray
multiUpdateGL(GLSC.ex[[2]][1], Version = "3.53.0")
1 hla#3.53.0#HLA-A*02:01:01:01~HLA-C*07:02:01:01~HLA-B*07:02:01:01~HLA-DRB1*1
5:01:01:01~HLA-DQB1*06:02:01:01~HLA-DPB1*04:02:01:01+HLA-A*01:01:01:01~HLA-C*
06:02:01:01~HLA-B*57:01:01:01~HLA-DRB1*07:01:01:01~HLA-DQB1*03:03:02:01~HLA-D
PB1*04:01:01:01
```

Translate vectors and data frames of HLA allele name data across IPD-IMGT/HLA Database Release Versions

The GIANT() function applies translateGLstring() to translate HLA allele name data across IPD-IMGT/HLA Database release versions, back to version 1.05.0, and functions on both vectors and data frames of HLA allele name data, including BIGDAWG-formatted HLA datasets. Data frames that are not BIGDAWG-formatted must be entirely composed of columns of HLA allele name data.

Parameters

- *data* A data frame or vector of HLA allele name character string data. Allele names in vectors must include locus prefixes. Data frames must include column headers identifying a single locus for the allele names in that column.
- *transFrom* A dot-formatted character string identifying the IPD-IMGT/HLA Database version (e.g. 3.59.0) under which the HLA data were generated.
- *transTo* A dot-formatted character string identifying the IPD-IMGT/HLA Database version (e.g. 2.25.0) to which which the HLA data are to be translated.

Translation of vectors of HLA allele names

```
GIANT(c("A*01:01:01:01","DQA1*01:01:01:01"),"3.56.0","2.20.0")
[1] "A*01010101"  "DQA1*010101"
GIANT(c("A*01:01:01:01","DQA1*01:01:01:01"),"3.56.0","3.00.0")
[1] "A*01:01:01:01" "DQA1*01:01:01"
GIANT(c("A*01:01:01:01","DQA1*01:01:01:01"),"3.56.0","1.09.0")
[1] "A*01011"   "DQA1*0101"
```

Translation of BIGDAWG-formatted HLA data

```
sHLAdata[1:5,c(1:8,15:18)]
   Subject Status           A         A.1           C         C.1           B
B.1
1 UT900-23      0        <NA>        <NA> 01:02:01:01    02:10:06 13:01:01:01
18:01:02
2 UT900-24      0 01:01:01:01 02:01:01:01 03:07:01:01       06:05 14:01:01:01
39:02:01:01
3 UT900-25      0       02:10    03:01:02       07:12 01:02:01:01       15:20
13:01:01:01
4 UT900-26      0 01:01:01:01       02:18 08:04:01:01    12:02:01 35:09:01:01
40:05:01:01
5 UT910-01      0 25:01:01:01 02:01:01:01 15:07:01:01 03:07:01:01    51:01:03
14:01:01:01

         DPA1      DPA1.1         DPB1      DPB1.1
1 02:01:01:01 02:01:01:01      103:01 14:01:01:01
2 01:03:01:01    02:01:03 14:01:01:01 14:01:01:01
3 01:03:01:01 01:03:01:01      103:01       58:01
```

```
4 01:03:01:01 01:03:01:01 14:01:01:01      103:01
5 01:03:01:01 02:01:01:01 14:01:01:01       58:01

GIANT(sHLAdata,"3.56.0","2.10.0")[1:5,c(1:8,15:18)]
   Subject Status        A      A.1      C    C.1      B    B.1   DPA1 DPA1.1
DPB1 DPB1.1
1 UT900-23      0     <NA>     <NA> 010201 020205   1301 180102 020101 020101
0403   1401
2 UT900-24      0 01010101 02010101   0307   0605   1401 390201 010301 020103
1401   1401
3 UT900-25      0     0210   030102   0712 010201   1520   1301 010301 010301
0403   5801
4 UT900-26      0 01010101     0218   0804 120201 350901   4005 010301 010301
1401   0403
5 UT910-01      0   250101 02010101   1507   0307 510103   1401 010301 020101
1401   5801

GIANT(sHLAdata,"3.56.0","1.15.0")[1:5,c(1:8,15:18)]
DPB1*103:01 has been removed from the Allele List or has had its name changed
.
DPB1*103:01 has been removed from the Allele List or has had its name changed
.
   Subject Status     A   A.1    C   C.1     B   B.1  DPA1 DPA1.1 DPB1 DPB1.1
1 UT900-23      0  <NA>  <NA> 0102 02025  1301 18012 02011  02011 <NA>   1401
2 UT900-24      0 01011 02011 0307  0605  1401 39021 01031  02013 1401   1401
3 UT900-25      0  0210 03012 0712  0102  1520  1301 01031  01031 <NA>   5801
4 UT900-26      0 01011  0218 0804 12021 35091  4005 01031  01031 1401   <NA>
5 UT910-01      0  2501 02011 1507  0307 51013  1401 01031  02011 1401   5801
```

In these examples, the DPB1*103:01 allele was originally named DPB1*04:03, but this allele was not identified until release version 2.0.0.

Convert GL String-formatted data to UNIFORMAT-formatted data

validateGLstring

The *validateGLstring()* function evaluates version 1.0 and 1.1 GL Strings for incorrect characters. TRUE is returned when all characters in the GL String are valid for the specified version. FALSE is returned when the GL String contains invalid characters for the version.

Parameters

- *GLstring* A character string of GL String-formatted HLA allele names and GL String 1.0 or 1.1 operators.
- *version* A character string of either ‘1.0’ or ‘1.1’ identifying the GL String version.

```
GLstring <- "HLA-A*02:01/HLA-A*02:02?HLA-A*03:01/HLA-A*03:02"
validateGLstring(GLstring,"1.0")
HLA-A*02:01/HLA-A*02:02?HLA-A*03:01/HLA-A*03:02 contains characters or operat
ors not permitted in GL String version 1.0.
```

```
[1] FALSE
validateGLstring(GLstring,"1.1")
[1] TRUE
```

GLStoUNI

The *GLStoUNI()* function converts genotype data from GL String format to UNIFORMAT format. Because the GL String '?' delimiter has no UNIFORMAT cognate, this function does not support the '?' delimiter or the GL String v1.1 format.

Parameters

- *GLstring* A character string of GL String-formatted HLA allele names and GL String 1.0 operators.
- *prefix* A string containing a gene-system prefix (default is "HLA-").
- *pre* A logical that indicates whether 'prefix' should be appended to the allele names (TRUE) or not (FALSE). The default value is FALSE.

```
GLStoUNI("HLA-A*02:01/HLA-A*02:02+HLA-A*03:01/HLA-A*03:02")
[1] "A*02:01,A*03:01|A*02:01,A*03:02|A*02:02,A*03:01|A*02:02,A*03:02"
```

multiGLStoUNI

The *multiGLStoUNI()* function converts GL string formatted data in data frames or vectors to uniformat format. If a vector is provided, all elements must be GL Strings. If a data frame is provided, the first column is assumed to contain identifiers and will not be converted; the remaining columns should contain GL strings.

Parameters

- *GLstringArray* A data frame or a vector of GL String 1.0 formatted data. If 'GLstringArray' is a data frame with more than one column, the first column should contain only identifiers. If 'GLstringArray' is a vector, it should contain only GL Strings.
- *prefix* A string containing a gene-system prefix (default is "HLA-").
- *pre* A logical that indicates whether 'prefix' should be appended to the allele names (TRUE) or not (FALSE). The default value is FALSE.

Return If 'GLstringArray' is a data frame, a data frame of UNIFORMAT values is returned. If 'GLstringArray' is a vector, a vector of UNIFORMAT values is returned.

Example GL String data frame

```
GLstring.ex[1:3,]
Relation    Gl.String
Subject    1 HLA-A*02:01~HLA-C*07:02~HLA-B*07:02~HLA-DRB1*15:01~HLA-DQB1*06:0
2~HLA-DPB1*04:02+HLA-A*01:01~HLA-C*06:02~HLA-B*57:01~HLA-DRB1*07:01~HLA-DQB1*
03:03~HLA-DPB1*04:01
Subject    2 HLA-A*03:01~HLA-C*07:01~HLA-B*49:01~HLA-DRB1*04:05~HLA-DQB1*03:0
```

```
2~HLA-DPB1*02:01+HLA-A*01:01~HLA-C*07:01~HLA-B*08:01~HLA-DRB1*13:01~HLA-DQB1*
06:03~HLA-DPB1*04:01
Subject    3 HLA-A*11:01~HLA-C*04:01~HLA-B*15:01~HLA-DRB1*04:01~HLA-DQB1*03:0
2~HLA-DPB1*04:02+HLA-A*03:01~HLA-C*08:02~HLA
```

Example GL String vectors

```
GLstring.ex[2][1:3,]
[1] "HLA-A*02:01~HLA-C*07:02~HLA-B*07:02~HLA-DRB1*15:01~HLA-DQB1*06:02~HLA-DP
B1*04:02+HLA-A*01:01~HLA-C*06:02~HLA-B*57:01~HLA-DRB1*07:01~HLA-DQB1*03:03~HL
A-DPB1*04:01"
[2] "HLA-A*03:01~HLA-C*07:01~HLA-B*49:01~HLA-DRB1*04:05~HLA-DQB1*03:02~HLA-DP
B1*02:01+HLA-A*01:01~HLA-C*07:01~HLA-B*08:01~HLA-DRB1*13:01~HLA-DQB1*06:03~HL
A-DPB1*04:01"
[3] "HLA-A*11:01~HLA-C*04:01~HLA-B*15:01~HLA-DRB1*04:01~HLA-DQB1*03:02~HLA-DP
B1*04:02+HLA-A*03:01~HLA-C*08:02~HLA-B*14:02~HLA-DRB1*13:02~HLA-DQB1*06:09~HL
A-DPB1*04:01"
```

Convert GL String to UNIFORMAT

```
multiGLStoUNI(GLstring.ex[1:5,1:2])
  Relation
Gl.String
1  Subject A*02:01~C*07:02~B*07:02~DRB1*15:01~DQB1*06:02~DPB1*04:02,A*01:01~C
*06:02~B*57:01~DRB1*07:01
~DQB1*03:03~DPB1*04:01
2  Subject A*03:01~C*07:01~B*49:01~DRB1*04:05~DQB1*03:02~DPB1*02:01,A*01:01~C
*07:01~B*08:01~DRB1*13:01
~DQB1*06:03~DPB1*04:01
3  Subject A*11:01~C*04:01~B*15:01~DRB1*04:01~DQB1*03:02~DPB1*04:02,A*03:01~C
*08:02~B*14:02~DRB1*13:02
~DQB1*06:09~DPB1*04:01
4  Subject A*68:01~C*15:02~B*40:06~DRB1*16:02~DQB1*05:02~DPB1*10:01,A*68:01~C
*06:02~B*45:01~DRB1*04:05
~DQB1*03:02~DPB1*11:01
5  Subject A*02:01~C*05:01~B*44:02~DRB1*15:01~DQB1*06:02~DPB1*02:01,A*30:01~C
*06:02~B*13:02~DRB1*07:01
~DQB1*02:02~DPB1*17:01

multiGLStoUNI(GLstring.ex[[2]][1:5])
[1] "A*02:01~C*07:02~B*07:02~DRB1*15:01~DQB1*06:02~DPB1*04:02,A*01:01~C*06:02
~B*57:01~
DRB1*07:01~DQB1*03:03~DPB1*04:01"
[2] "A*03:01~C*07:01~B*49:01~DRB1*04:05~DQB1*03:02~DPB1*02:01,A*01:01~C*07:01
~B*08:01~
DRB1*13:01~DQB1*06:03~DPB1*04:01"
[3] "A*11:01~C*04:01~B*15:01~DRB1*04:01~DQB1*03:02~DPB1*04:02,A*03:01~C*08:02
~B*14:02~
DRB1*13:02~DQB1*06:09~DPB1*04:01"
[4] "A*68:01~C*15:02~B*40:06~DRB1*16:02~DQB1*05:02~DPB1*10:01,A*68:01~C*06:02
```

```
~B*45:01~
DRB1*04:05~DQB1*03:02~DPB1*11:01"
[5] "A*02:01~C*05:01~B*44:02~DRB1*15:01~DQB1*06:02~DPB1*02:01,A*30:01~C*06:02
~B*13:02~
DRB1*07:01~DQB1*02:02~DPB1*17:01"
```

Convert UNIFORMAT-formatted data to GL String-formatted data

validateUniformat

The *validateUniformat()* function evaluates UNIFORMAT data for incorrect characters. TRUE is returned when all characters in the UNIFORMAT data are valid for the specified version. FALSE is returned when the UNIFORMAT data contain invalid characters.

Parameters

- *uniformat* A character string of allele names and operators in the UNIFORMAT format.

```
validateUniformat("A*02:01,A*03:01|A*02:01,A*03:02|A*02:02,A*03:01|A*02:02,A*
03:02")
[1] TRUE

validateUniformat("A*02:01+A*03:01|A*02:01,A*03:02|A*02:02,A*03:01|A*02:02,A*
03:02")
A*02:01+A*03:01|A*02:01,A*03:02|A*02:02,A*03:01|A*02:02,A*03:02 contains oper
ators or characters not permitted in UNIFORMAT.
[1] FALSE
```

UNItoGLS

The *UNItoGLS()* function converts a single UNIFORMAT string to GL String format.

Parameters

- *uniformat* A string of HLA allele names and operators in the UNIFORMAT format signifying their relation with one another.
- *prefix* A character string of the desired gene-system prefix (default is "HLA-").
- *pre* A logical that indicates whether returned allele names should contain 'prefix' (TRUE), or if 'prefix' should be excluded from returned names (FALSE).

```
UNItoGLS("A*02:01,A*03:01|A*02:01,A*03:02|A*02:02,A*03:01|A*02:02,A*03:02")
[1] "HLA-A*02:01/HLA-A*02:02+HLA-A*03:01/HLA-A*03:02"
```

multiUNItoGLS

The *multiUNItoGLS()* function translates columns of arrays from uniformat format to GL string format. If one column is entered, it must be in UNIFORMAT format. If multiple columns are entered, the first column (furthest left) should contain an identification factor

of some kind, as the first column will not be translated, and the remaining columns should contain UNIFORMAT strings. Examples of input formats are presented below.

Example UNIFORMAT Data Frame

```
> UNIFORMAT.example[1:3,]
   sample.id  genotype
1 hhrv_id195  blank,A*02:01|A*02:01,A*02:01 blank,B*44:03|B*44:03,B*44:03 bla
nk,DRB1*07:01|DRB1*07:01,DRB1*07:01
2 hhrv_id454  A*02:01,A*23:01|A*02:01,A*23:17 B*27:05,B*44:03|B*27:13,B*44:03
DRB1*07:01,DRB1*11:04
3 hhrv_id642  A*66:01,A*31:08|A*31:08,A*66:08 B*27:05,B*07:02|B*27:05,B*07:61
|B*27:13,B*07:02|B*27:13,B*07:61 DRB1*01:01,DRB1*16:02
```

Example UNIFORMAT Vector

```
> UNIFORMAT.example[2][1:3,]
[1] "blank,A*02:01|A*02:01,A*02:01 blank,B*44:03|B*44:03,B*44:03 blank,DRB1*0
7:01|DRB1*07:01,DRB1*07:01"
[2] "A*02:01,A*23:01|A*02:01,A*23:17 B*27:05,B*44:03|B*27:13,B*44:03 DRB1*07:
01,DRB1*11:04"
[3] "A*66:01,A*31:08|A*31:08,A*66:08 B*27:05,B*07:02|B*27:05,B*07:61|B*27:13,
B*07:02|B*27:13,B*07:61 DRB1*01:01,DRB1*16:02"
```

Parameters

- *UniformatArray* A data frame or vector of UNIFORMAT formatted strings.
- *prefix* A character string of the desired gene-system prefix (default is "HLA-").
- *pre* A logical that indicates whether returned allele names should contain 'prefix' (TRUE), or if 'prefix' should be excluded from returned names (FALSE).

Return If 'uniformat'is a data frame, a data frame of GL String values is returned. If 'uniformat' is a vector, a vector of GL String values is returned.

Convert UNIFORMAT to GL String

```
multiUNItoGLS(UNIFORMAT.example[1:3,])
   sample.id
genotype
1 hhrv_id195       HLA-blank/HLA-A*02:01+HLA-A*02:01^HLA-blank/HLA-B*44:03+HL
A-B*44:03^HLA-blank/HLA-DRB1*07:01+HLA-DRB1*07:01
2 hhrv_id454             HLA-A*02:01+HLA-A*23:01/HLA-A*23:17^HLA-B*27:05/HLA-
B*27:13+HLA-B*44:03^HLA-DRB1*07:01+HLA-DRB1*11:04
3 hhrv_id642 HLA-A*66:01/HLA-A*66:08+HLA-A*31:08^HLA-B*27:05/HLA-B*27:13+HLA-
B*07:02/HLA-B*07:61^HLA-DRB1*01:01+HLA-DRB1*16:02

multiUNItoGLS(UNIFORMAT.example[2][1:3,])
[1] "HLA-blank/HLA-A*02:01+HLA-A*02:01^HLA-blank/HLA-B*44:03+HLA-B*44:03^HLA-
blank/HLA-DRB1*07:01+HLA-DRB1*07:01"
[2] "HLA-A*02:01+HLA-A*23:01/HLA-A*23:17^HLA-B*27:05/HLA-B*27:13+HLA-B*44:03^
```

```
HLA-DRB1*07:01+HLA-DRB1*11:04"
[3] "HLA-A*66:01/HLA-A*66:08+HLA-A*31:08^HLA-B*27:05/HLA-B*27:13+HLA-B*07:02/
HLA-B*07:61^HLA-DRB1*01:01+HLA-DRB1*16:02"
```

Convert GL String to UNIFORMAT

```
multiGLStoUNI(GLstring.ex[1:5,1:2])
  Relation
Gl.String
1  Subject A*02:01~C*07:02~B*07:02~DRB1*15:01~DQB1*06:02~DPB1*04:02,A*01:01~C
*06:02~B*57:01~DRB1*07:01
~DQB1*03:03~DPB1*04:01
2  Subject A*03:01~C*07:01~B*49:01~DRB1*04:05~DQB1*03:02~DPB1*02:01,A*01:01~C
*07:01~B*08:01~DRB1*13:01
~DQB1*06:03~DPB1*04:01
3  Subject A*11:01~C*04:01~B*15:01~DRB1*04:01~DQB1*03:02~DPB1*04:02,A*03:01~C
*08:02~B*14:02~DRB1*13:02
~DQB1*06:09~DPB1*04:01
4  Subject A*68:01~C*15:02~B*40:06~DRB1*16:02~DQB1*05:02~DPB1*10:01,A*68:01~C
*06:02~B*45:01~DRB1*04:05
~DQB1*03:02~DPB1*11:01
5  Subject A*02:01~C*05:01~B*44:02~DRB1*15:01~DQB1*06:02~DPB1*02:01,A*30:01~C
*06:02~B*13:02~DRB1*07:01
~DQB1*02:02~DPB1*17:01

multiGLStoUNI(GLstring.ex[[2]][1:5])
[1] "A*02:01~C*07:02~B*07:02~DRB1*15:01~DQB1*06:02~DPB1*04:02,A*01:01~C*06:02
~B*57:01~
DRB1*07:01~DQB1*03:03~DPB1*04:01"
[2] "A*03:01~C*07:01~B*49:01~DRB1*04:05~DQB1*03:02~DPB1*02:01,A*01:01~C*07:01
~B*08:01~
DRB1*13:01~DQB1*06:03~DPB1*04:01"
[3] "A*11:01~C*04:01~B*15:01~DRB1*04:01~DQB1*03:02~DPB1*04:02,A*03:01~C*08:02
~B*14:02~
DRB1*13:02~DQB1*06:09~DPB1*04:01"
[4] "A*68:01~C*15:02~B*40:06~DRB1*16:02~DQB1*05:02~DPB1*10:01,A*68:01~C*06:02
~B*45:01~
DRB1*04:05~DQB1*03:02~DPB1*11:01"
[5] "A*02:01~C*05:01~B*44:02~DRB1*15:01~DQB1*06:02~DPB1*02:01,A*30:01~C*06:02
~B*13:02~
DRB1*07:01~DQB1*02:02~DPB1*17:01"
```

---

5. Data Analysis Tools

The functions described in this section either perform data analyses or generate specific data sets suitable for additional analyses. All consume BIGDAWG-formatted datasets.

Example Data

To facilitate testing and experimentation with these functions, the 'sHLAdata' data object is included in the package. This BIGDAWG-formatted HLA genotype data object represents completely synthetic IPD-IMGT/HLA release 3.56.0 HLA-A, -C, -B, -DRB1, -DQA1, -DQB1, -DPA1, and -DPB1 genotype data for 24 control subjects and 23 case subjects, and does not represent any true human population.

Calculating Relative Risk with BIGDAWG-formatted Datasets

relRisk

The relRisk() function calculates relative risk (RR) for individual alleles and genotypes in BIGDAWG-formatted datasets. RR analysis is intended for *non-case-control* datasets. While two subject categories (0 and 1) are required in the **Subject** column of the BIGDAWG-formatted datafile, the categories should *not* be patients and controls. Instead, the categories may be, e.g., two groups of patients with the same disease, where one group has more severe symptoms (coded as 1), and other has mild symptoms (coded as 0). In this case, relRisk() identifies the risk of sefverity associated with a given allele or genotype.

relRisk() returns a list object of two lists ("alleles" and "genotypes"), each of which contains relative risk, confidence interval and p-value data for the individual alleles and individual genotypes at each locus in a BIGDAWG-formatted non-case-control genotype data frame or tab-delimited text file.

Parameters

- *dataset* The name of a non-case-control genotype dataset using the BIGDAWG format.
- *return* A logical identifying if the list object should be returned (return=TRUE), or if pairs of tab-delimited text files of results (one for alleles and one for genotypes) should be written to the working directory for each locus.
- *save.path* A character string identifying the path in which to write the pair of files when return is FALSE. The default value is tempdir().

```
rr <- relRisk(sHLAdata[,1:4])
```

Column headers in each returned data frame are, Locus, Variant, Status_1, Status_0, RelativeRisk, CI.low, CI.high, p.value, and Significant.

Division of BIGDAWG-formatted Datasets for Stratification Analyses

BDstrat: Multilocus Allele Stratification for BIGDAWG Datasets

The BDstrat() function divides a BIGDAWG-formatted] dataset into two strata, one of which contains all subjects with any of a set of specified alleles, while the other contains all

subjects without any of the specified alleles. If the positive-stratum and negative-stratum datasets have sufficient numbers of case and control (or exposed and unexposed) subjects, each stratum can be analyzed via the BIGDAWG package or relRisk(). Sample size is an important consideration when determining how many alleles on which to stratify.

Parameters

- *dataset*: A BIGDAWG-formatted data frame or a path to a BIGDAWG-formatted, tab-delimited text file.
- *alleles*: A vector of allele names in the * format (e.g., “A*01:01:01:01” for HLA alleles or “KIR2DL1*001” for KIR alleles).
- *warnBelow* : An integer that defines a low number of subjects in a stratum, generating a warning message. The default value is 21.

Return A list-object of two BIGDAWG-formatted data frames titled ‘dataset’$‘alleles’-positive’ and ‘dataset’$‘alleles’-negative` . The positive list element includes all subjects with the specified alleles, and the negative list element includes all subjects without those specified alleles.

```
Stratify on a single allele in the HLA_data dataset bundled with BIGDAWG
HLA_data.single.strat <- BDstrat(sHLAdata,"DRB1*08:02:01:01")

Stratify on three alleles at two loci in the HLA_data dataset bundled with BI
GDAWG
HLA_data.multi.strat <- BDstrat(sHLAdata,c("DRB1*04:02:01","DRB1*04:07:01:01"
,"A*32:04"))

Run BIGDAWG() on both multi-allele strata
for(i in 1:2) {BIGDAWG(HLA_data.multi.strat[[i]],HLA = TRUE,Run.Tests = "L")}
```

Conversion of BIGDAWG-formatted Datasets to *Python for Population Genetics* (PyPop)-formatted Datasets

BDtoPyPop : Enabling Population Genetic Analyses of Case and Control Datasets

- BDtoPyPop() converts a BIGDAWG-formatted case-control dataset into two PyPop version 1.*.* datasets – one for all ‘case’ subjects and one for all ‘control’ subjects. PyPop datasets can be exported as a pair of “.pop” files for analysis, or can be generated as a list object containing two data frames for additional manipulation before analysis.

Parameters

- *dataset* A BIGDAWG-formatted data frame containing a case-control dataset.
- *filename* A character string identifying either the desired path and name for the generated files, or the elements of the returned list object.

- *save.file* A logical indicating if the pair of files should be written (TRUE) or if a list object should be returned. The default value is TRUE.
- *save.path* A character string identifying the path in which to write the pair of files when return is FALSE. The default value is tempdir().

Return When save.file = TRUE a pair of PyPop-formatted text files named "'filename'.positive.pop" and "'filename'.negative.pop" are generated in the specified directory. The "'filename'.positive.pop" file includes all subjects with a value of 1 in the second column of the BIGDAWG data file, and the "'filename'.negative.pop" file includes all subjects with a value of 0 in the second column of the BIGDAWG data file.

When save.file = FALSE, a list-object of two PyPop-formatted data frames titled "'filename'.positive" and "'filename'-negative" is returned. The positive list element includes all subjects a value of 1 in the second column of the BIGDAWG data file, and the negative list element includes all subjects with a value of 0 in the second column of the BIGDAWG data file.

Note PyPop is not an R package. In order to analyze the files generated by this function with PyPop version 1.*.*, the pypop-genomics package must be installed on your system, and a PyPop configuration file, detailing the analyses to be performed, is required. A PyPop configuration file is not generated by BDtoPyPop().

```
Generating a list object of two PyPop datasets from the HLA_data dataset bund
led with BIGDAWG.
HLAdata.PP <- BDtoPyPop(sHLAdata,"BDHLA",FALSE)

Generating a pair PyPop data files from the HLA_data dataset bundled with BIG
DAWG.
BDtoPyPop(sHLAdata,"BDHLA",TRUE)
```

END OF VIGNETTE
