# Supplementary material for: HLAtools, Searching Shared HLA Amino Acid Residue Prevalence, and the Global Frequency Browsers: New Computational Resources for Working With HLA Data and Visualizing Global Patterns of HLA Variation
Source: Int J Immunogenet. 2025 Sep 14;52(6):358–70. doi: 10.1111/iji.70013 (PMC12595587; doi:10.1111/iji.70013)
Supplement: Supplementary file 2 — Supporting File 2: iji70013‐sup‐0002‐SuppMat.pdf [file IJI-52-358-s003.pdf]

SSHAARP: Searching Shared HLA Amino Acid Residue Prevalence

Livia Tran - livia.tran@uscf.edu

June 17, 2025

- Package Version: 2.0.8

Overview

The Searching Shared HLA Amino Acid Residue Prevalence (SSHAARP) Package processes alignment files produced by the IPD-IMGT/HLA Database to identify user-defined amino acid residue motifs across HLA alleles, HLA alleles, or HLA haplotypes, and calculates frequencies of the selected variant based on HLA allele frequency or haplotype data.

SSHAARP's functions interact with protein alignments from the ANHIG/IMGTHLA Github repository (https://github.com/ANHIG/IMGTHLA/tree/Latest/alignments). The first time PALM() is executed for a given locus, regardless of the variant type, PALM() will build the protein alignments for the locus in the provided variant. The locus specific alignment will be output to the temp directory. PALM() will check the temp directory for the locus of the entered variant; if the locus specific alignment exists, it will be loaded into the local environment for reference. Otherwise, the locus specific alignment will be built and output to the temp directory. Alignments will be available for PALM to reference until the R session is restarted. Once the R session is restarted, alignments will be regenerated with subsequent uses of PALM.

SSHAARP also interacts with the Solberg dataset, a user provided allele dataset formatted like the Solberg dataset, a mock haplotype dataset, or a user provided dataset formatted like the mock haplotype dataset. SSHAARP's functions also interact with the Generic Mapping Tools (gmt) R package. The Solberg dataset and mock haplotype dataset are included in the package. Details about the Solberg dataset can be found at doi: 10.1016/j.humimm.2008.05.001. The bundled solberg_dataset object is the 1-locus-alleles.dat file in the results.zip archive at http://pypop.org/popdata/.

The gmt R package requires operating system installations of GMT software (version 5 or 6; available at: https://www.soest.hawaii.edu/gmt/) and Ghostscript, version 9.6 or higher (https://ghostscript.com/releases/gsdnld.html), to generate maps. SSHAARP v 2.0.1 uses GMT v 6.5.0. GMT v 6.5.0 was used to create the maps included in the vignette. The installer can be downloaded from the GMT website. Linux and Mac OSx users have the option of installing via homebrew. Run `brew install gmt` for the most recent, stable installation of GMT. Ghostscript can also be installed with homebrew by running `brew install ghostscript`. Earlier versions of Ghostscript may result in the generation of incorrect maps. To determine if maps are being generated correctly, compare maps for the `DRB1*26F~28E~30Y` motif to the examples in this vignette.

Functions

PALM()

PALM() generates a frequency heatmap for an HLA allele, HLA amino-acid motif, or HLA haplotype. Alleles and amino acid motif maps are generated based on the allele frequency data in the Solberg dataset, or the user provided dataset. Haplotypes maps are generated based on the mock haplotype dataset, or the user

specified dataset. User specified datasets MUST follow the same format and convention as the Solberg Dataset or mock haplotype dataset. Maps are created using the gmt R package and the Generic Mapping Tools (GMT) map-making software. GMT software is required for this function and can be downloaded at https://www.soest.hawaii.edu/gmt/.

JPEG heatmap files named " 'variant'.jpg", where 'variant' is an allele, motif, or haplotype, are written to a directory identified by the direct parameter, with the working directory set as the default. The file names of maps generated on Microsoft Windows systems have a different delimiter between the locus name and the amino acid positions; instead of an asterisk (*), a dash (-) is used to accommodate for Windows naming conventions (e.g. `DRB1-26F~28E~30Y`).

The `filename` parameter is either the full path to the user specified dataset, the Solberg dataset bunded in the package, or the mock haplotype dataset bundled in the package.

The `variantType` parameter identifies whether the variant entered is an allele, motif, or haplotype. The `variantType` parameter must be changed to 'allele' if an allele frequency map is desired, or changed to 'haplotype' if a haplotype frequency map is desired. Alleles in haplotypes may be delimited with "-" or "~".

The `mask` parameter defaults to FALSE, where all areas on the generated map are visible. If `mask = TRUE`, areas with little to no data table coverage will be masked out.

The `color` and `filter_migrant` parameters default to TRUE. When `color = TRUE`, PALM generates a color heatmap; when `color = FALSE`, a greyscale heatmap is generated. When `filterMigrant = TRUE`, populations from the OTH region and populations with complexity values suffixed with 'mig', or migrant populations, are excluded from the heatmap plot. When `filter_migrant = FALSE`, these populations are included in the heatmap plot.

The `mapScale` parameter determines if the max frequency of the map scale should be 1, or the max frequency of the chosen motif, allele, or haplotype. When `mapScale = TRUE`, which is the default, the max frequency of the chosen variant is used. When mapScale=FALSE, the max frequency is 1.

The `direct` parameter is the working directory to output created maps to. The default is the user's current working directory.

The `generateLowFrequency` parameter determines whether maps should be generated for a variant if the maximum frequency for the variant is low frequency. Low frequency populations are defined as those with a frequency of 0.000, indicating three zeros after the decimal point.

The `resolution` parameter is an integer value for raster resolution in dpi for the final map output. It is not recommended to go below 400. Default is set to 500.

If an allele with more than two fields is entered and the Solberg dataset is selected as the data source, the allele will automatically be truncated to two fields, as the Solberg dataset only contains two field alleles.

The Ghostscript software suite (>= version 9.26) is required to generate the heatmap file. Versions of Ghostscript < 9.26 introduce a layering error during heatmap generation. To determine if maps are being generated correctly, compare heatmaps of the `DRB1*26F~28E~30Y` motif (with filter_migrant = TRUE) against the examples in this vignette.

Note: While the map legend identifies the highest frequency value, values in this range may not be represented on the map due to frequency averaging over neighboring populations.

```
#color motif heatmap plot with masking OFF
>PALM("DRB1*26F~28E~30Y", variantType="motif", filename=SSHAARP::solberg_dataset, resolution = 480)
```

```
#greyscale allele heatmap plot with masking OFF
> PALM("DRB1*01:01", variantType="allele", color=F, mask = T, filename=SSHAARP::solberg_dataset, resolu
```

DRB1*26F~28E~30Y

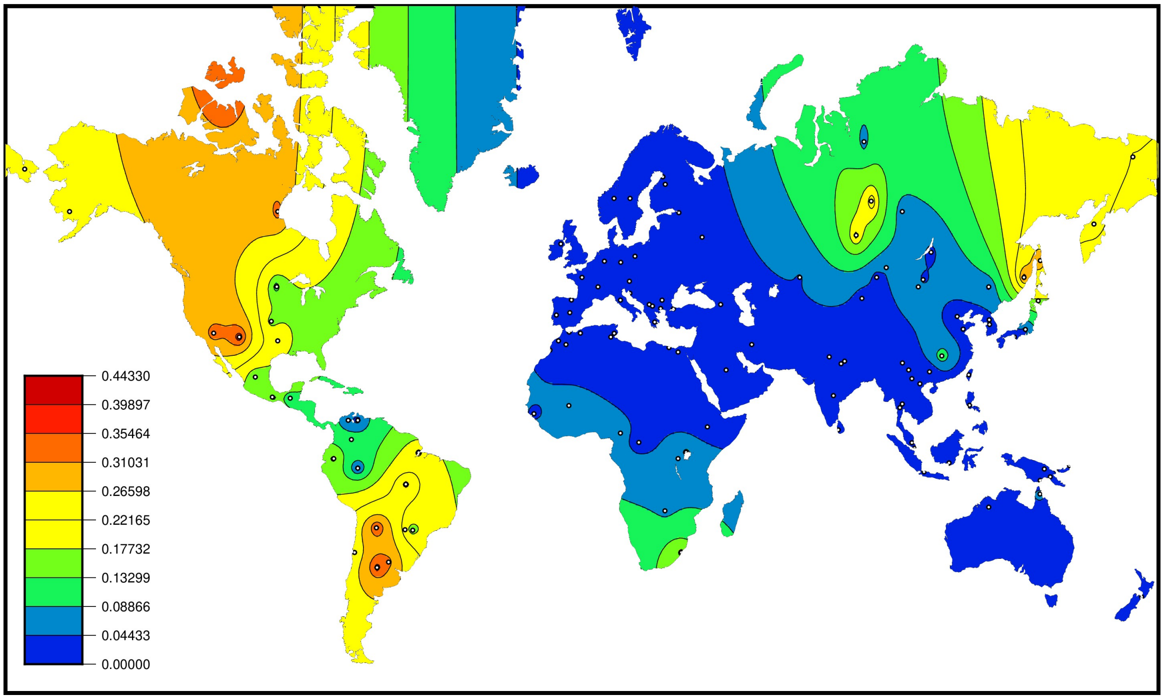

Figure 1: ...

DRB1*01:01

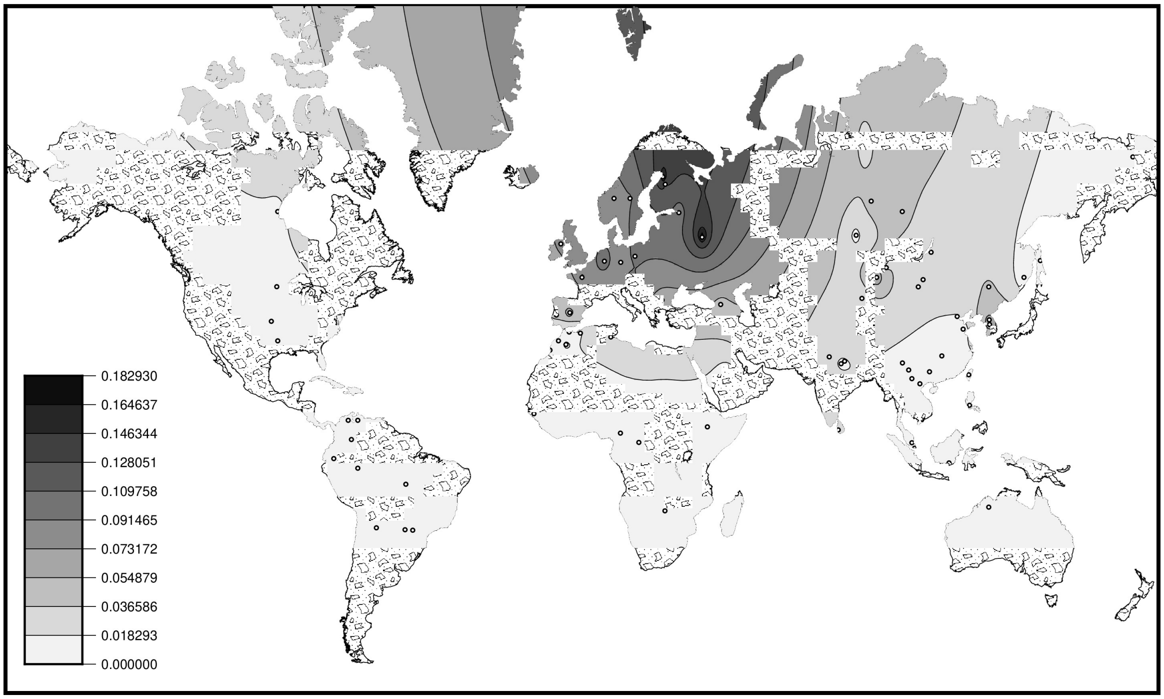

Figure 2: ...

A*01:01~DRB1*01:01

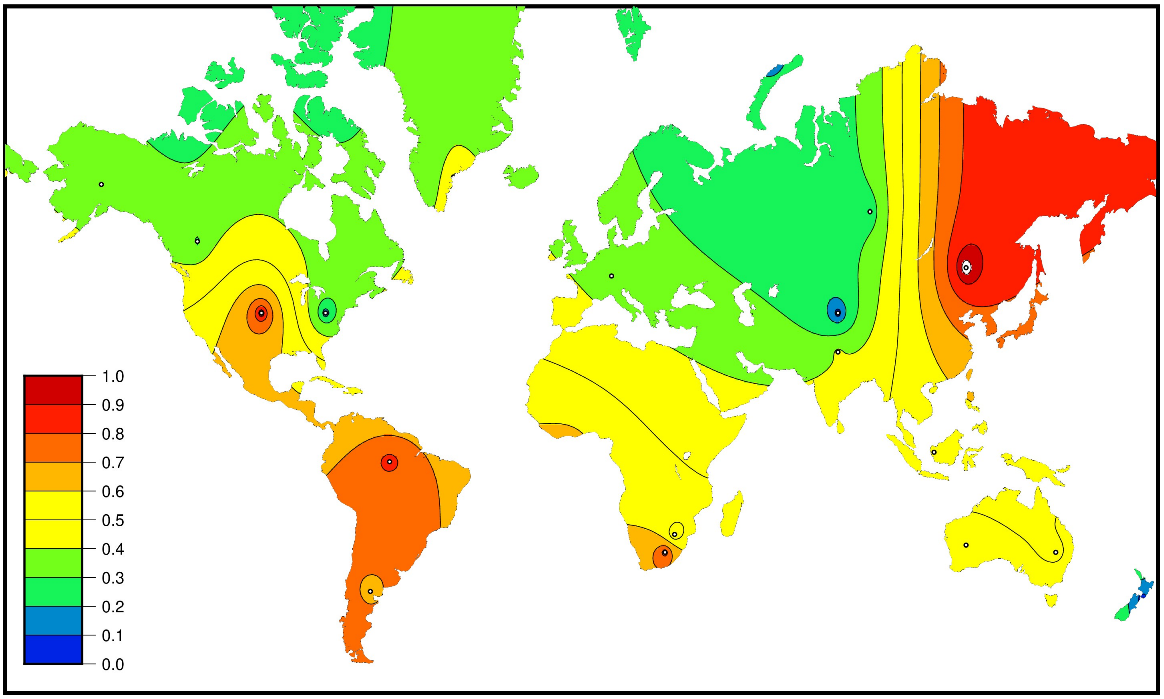

Figure 3: ...

```
#color haplotype heatmap plot with masking OFF with bundled mock haplotype dataset
PALM("A*01:01~DRB1*01:01", variantType="haplotype", filename=SSHAARP::mock_haplotype_dataset, color = TI


#if the locus of a variant is valid but not in the Solberg dataset, a warning message is output:
> PALM("TAP1*01:01", variantType="allele", filename=SSHAARP::solberg_dataset)

Warning message:
In PALM("TAP1*01:01", variantType = "allele", filename=SSHAARP::solberg_dataset) :
  TAP1 is a valid locus, but is not in the Solberg dataset

#if an amino acid motif does not exist, a warning is output:
>PALM('A*-24F', variantType = "motif", filename=SSHAARP::solberg_dataset)

Warning message:
In PALM("A*-24F", variantType = "motif", filename = SSHAARP::solberg_dataset) :
  A*-24F: No alleles possess this motif

#if an amino acid motif exists but is not found in the Solberg dataset, a warning is output:
>PALM("DRB1*-25R~13S~16H~26Y~47Y~86V", variantType="motif", filename=SSHAARP::solberg_dataset)

Warning message:
In PALM("DRB1*-25R~13S~16H~26Y~47Y~86V", variantType="motif", filename=SSHAARP::solberg_dataset) :
  DRB1*-25R~13S~16H~26Y~47Y~86V: No alleles possess this motif

#if an allele is not found in IMGTprotalignments, a warning is output:
> PALM("DRB1*01999:01", variantType="allele",filename=SSHAARP::solberg_dataset)

Warning message:
In PALM("DRB1*01999:01", variantType = "allele", filename=SSHAARP::solberg_dataset) :
  The allele you entered is not present in the current release of ANHIG/IMGTHLA alignments.

#if an allele is valid but is not present in the Solberg dataset, a warning is output:
> PALM("A*01:37", variantType = "allele", filename=SSHAARP::solberg_dataset)

Warning message:
In PALM("A*01:37", variantType = "allele", filename=SSHAARP::solberg_dataset) :
  The A*01:37 allele entered is a valid allele and exists in the IMGT protein alignments, but is not pr
```
