# Supplementary material for: HLAtools, Searching Shared HLA Amino Acid Residue Prevalence, and the Global Frequency Browsers: New Computational Resources for Working With HLA Data and Visualizing Global Patterns of HLA Variation
Source: Int J Immunogenet. 2025 Sep 14;52(6):358–70. doi: 10.1111/iji.70013 (PMC12595587; doi:10.1111/iji.70013)
Supplement: Supplementary file 3 — Supporting File 3: Locations of HLA‐Y and HLA‐R in the Class I Region. DRB Gene Locations on Six Human Genome Reference Assemblies. [file IJI-52-358-s005.pdf]

Figure S1. Locations of *HLA-Y* and *HLA-R* in the Class I Region

Legend: The positions of the *HLA-W*, *MICD* and *HLA-J* pseudogenes, relative to their distance from the 5' end of the *HLA-W* gene on the hg38 (GRCh38.p14 [GCF_000001405.40]) reference assembly NC_000006.12, are presented in the lower section of the figure.

The positions of the *HLA-Y* and *HLA-R* pseudogenes, relative to their distance from the 5' end of a 60,145 kb insertion between the *MICD* and *HLA-J* genes, are shown in the upper section. The green triangle identifies the position of the insertion (Alexandrov et al. 2023), 30,946 nucleotides from the 5' end of *HLA-W*. The upper and lower sections of the figure use the same distance scale.

Arrows indicate the genomic strand (Watson/reference [+] or Crick/complementary [-]) on which the gene is read; arrows for genes on the + strand point to the right, while arrows for genes on the – strand point to the left. The coding strands for *HLA-Y* and *HLA-R* have not been described. The lengths of the arrows and boxes represent the relative gene lengths (within 1% of the actual length).

Figure S2. *DRB* Gene Locations on Six Human Genome Reference Assemblies

Legend: The positions of the nine *HLA-DRB* genes are presented relative to their distance from the 5' start sites of *HLA-DRA* genes on six hg38 (GRCh38.p14 [GCF_000001405.40]) human genome reference assemblies. Accession identifiers for the reference genome (NC_000006.12) and five contigs (NT_*) are presented on the left.

DRA genes are illustrated in grey. Each *DRB* gene is identified with a unique color. Expressed *DRB* genes are illustrated in shades of blue, and *DRB* pseudogenes in shades of red. *DRA* genes are identified with the letter *A*, and *DRB* genes with their corresponding gene numbers (e.g., *DRB1* genes are identified with "1"). Full gene names are presented in the box at the bottom of the figure.

Arrows indicate the genomic strand (Watson/reference [+] or Crick/complementary [-]) on which the gene is read; arrows for genes on the + strand point to the right, while arrows for genes on the – strand point to the left. The lengths of the arrows represent the relative gene lengths (within 1% of the actual length). A 14,968-nucleotide section of unreported sequence in the NT_167246.2 contig is identified as "15kb".
