# Supplementary figures and images for: HLAtools, Searching Shared HLA Amino Acid Residue Prevalence, and the Global Frequency Browsers: New Computational Resources for Working With HLA Data and Visualizing Global Patterns of HLA Variation
Source: Int J Immunogenet. 2025 Sep 14;52(6):358–70. doi: 10.1111/iji.70013 (PMC12595587; doi:10.1111/iji.70013)

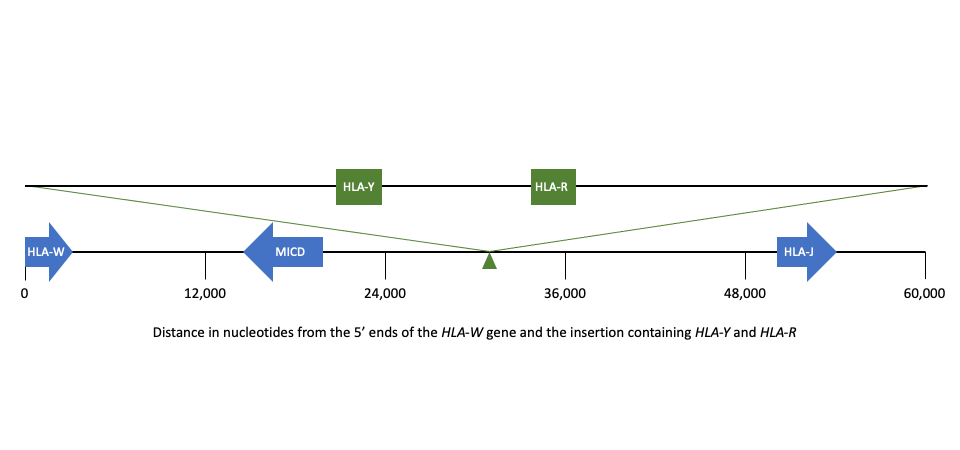

Supplement: Supplementary file 4 — Supporting File 4: iji70013‐sup‐0005‐figureS1.png [file IJI-52-358-s002.png]

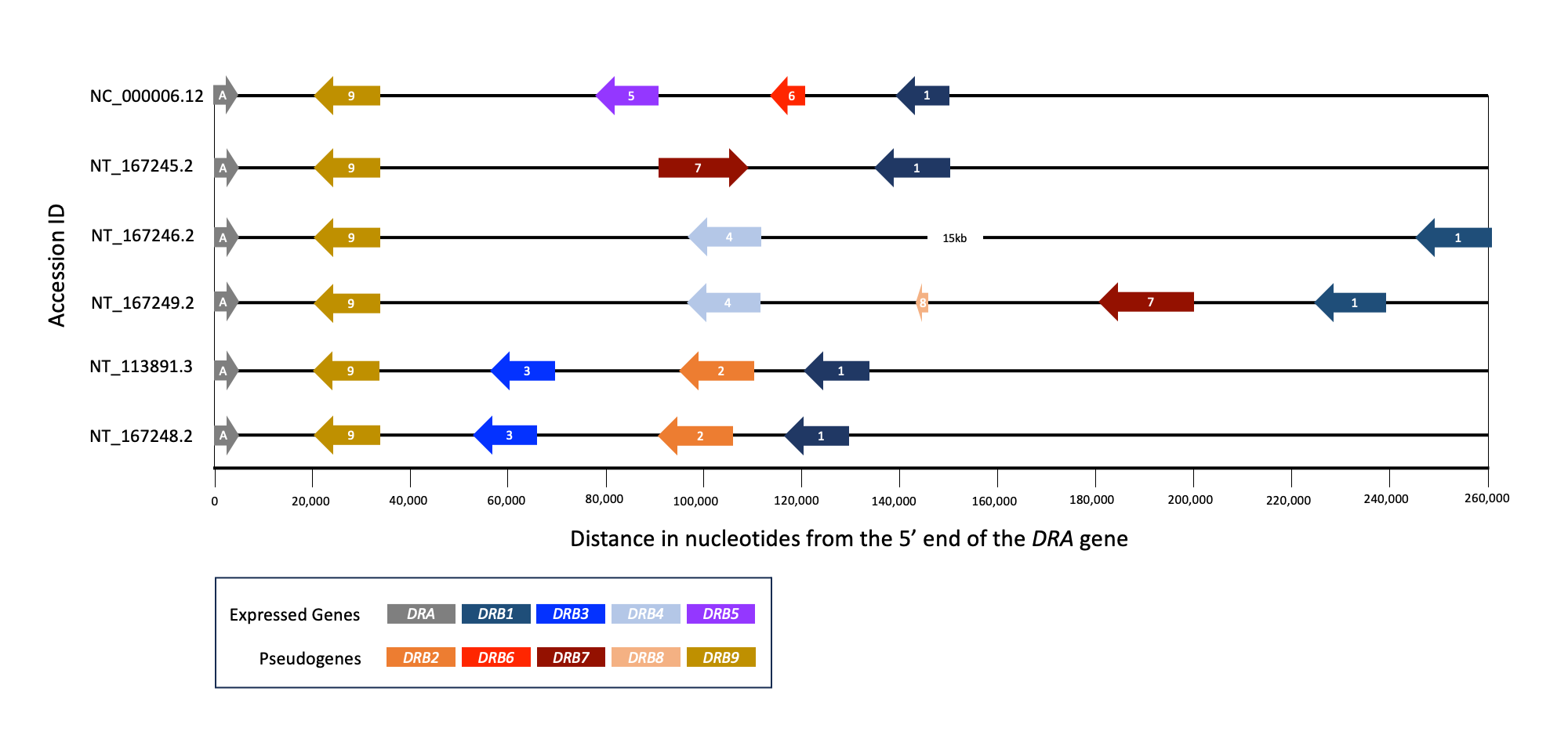

Supplement: Supplementary file 5 — Supporting File 5: iji70013‐sup‐0005‐figureS2.png [file IJI-52-358-s004.png]
